# Carbon-11 Isotopic Radiolabeling of CP31398 and Development of a Fluorine-18 Derivative to Target Protein p53 with PET Imaging

**DOI:** 10.1021/acsomega.5c06415

**Published:** 2026-01-28

**Authors:** Sébastien Beuché, Soizic Martin Aubert, Philippe Robin, Caroline Denis, Denis Servent, Bertrand Kuhnast, Charles Truillet, Fabien Caillé

**Affiliations:** † Université Paris-Saclay, Inserm, CNRS, CEA, Laboratoire d’Imagerie Biomédicale Multimodale Paris-Saclay (BioMaps), 4 place du Général Leclerc 91401 Orsay, France; ‡ Université Paris-Saclay, CEA, Département Médicaments et Technologies pour la Santé (DMTS), SIMoS, 91191 Gif sur Yvette, France

## Abstract

Mutant protein p53, a central driver of pro-oncological deregulations, is widely recognized as a biomarker of cancer aggressiveness and therapy resistance. In a personalized medicine perspective, positron emission tomography (PET) imaging of mutant p53 would be a powerful tool for patient stratification and drug development. However, to date, no PET radiotracers directly targeting mutant p53 have been reported. Inspired by the CP31398 drug, which stabilizes p53 conformation and treats tumors expressing mutant p53, we designed two novel PET radiotracers labeled with either carbon-11 by isotopic labeling or fluorine-18, namely [^11^C]**CP31398** and [^18^F]**FG-CP31398**. The nonradioactive fluorinated analogue was synthesized, as well as the two radiolabeling precursors. Optimization of the radiomethylation with carbon-11 of the phenol precursor was achieved, and automated radiosynthesis of [^11^C]**CP31398** afforded the ready-to-inject radiotracer with 40 ± 13% radiochemical yield and 39 ± 12 GBq/μmol (*n* = 6) molar activity after quality control. Automated radiofluorination with ^18^F-fluoride by aliphatic S_N_2 of a tosylate precursor afforded the ready-to-inject [^18^F]**FG-CP31398** in 10 ± 4% radiochemical yield (RCY) and 80 ± 39 GBq/μmol (*n* = 4) molar activity after quality control. Binding experiments performed with [^18^F]**FG-CP31398** on HEK-293T cells transfected with a green fluorescent protein-p53 wild-type plasmid, with or without presaturation with **CP31398**, demonstrated that despite the chemical modification performed on this compound, [^18^F]**FG-CP31398** was still able to bind specifically to the cell with *ca.* 20% nonspecific binding. However, autoradiography experiments performed after the incubation of either [^11^C]**CP31398** or [^18^F]**FG-CP31398** on H358 (p53 positive) and A549 (p53 negative) tumor slices derived from human lung cancer cells revealed that both tracers were not able to bind the p53-positive cells. Surprisingly, a specific fixation demonstrated by presaturation experiments was observed for both radiotracers on the p53-negative A549 cells. Overall, our findings indicate that neither **CP31398** nor **FG-CP31398** binds directly to p53 but instead interacts with an unidentified target. While unsuitable for p53 imaging, these radiotracers may serve as valuable tools to unravel the controversial mechanism of action of CP31398.

## Graphical abstract

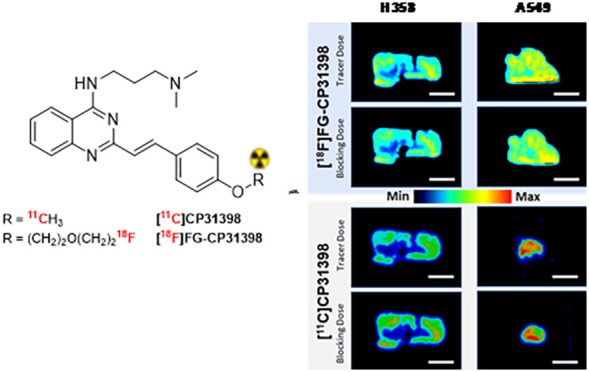

## Introduction

The protein p53, also known as “tumor protein p53”, is an ubiquitous nuclear protein which regulates DNA transcription.^1^ p53 is a 53 kDa protein composed of six domains, including a DNA-binding domain and an oligomerization domain, essential for its transcriptional activity.^2^ As the guardian of the genome, p53 is responsible for eliminating premalignant cells by inducing cell cycle arrest, apoptosis, or senescence.^3^ While in normal cells p53 is maintained at low levels by different regulators like murine double minute 2 (MDM2), an ubiquitin ligase,^4^ the protein is stabilized in response to various cellular stresses, including DNA damages or chromothripsis, triggering either DNA repair mechanisms or cell death.

In over 50% of cancers, tumor cells present mutations of the TP53 gene encoding for p53, leading to structural modifications of its DNA-binding domain, thus altering the repair and protection functions of the protein.^5,6^ Germline mutations of TP53 are associated with the Li-Fraumeni multicancer predominance syndrome, and missense mutations in the central DNA-binding domain of the protein trigger various cancers (lung, prostate, breast, brain, sarcoma, leukemia, etc.) with etiology linked to the mutational spectrum.^7^ Moreover, mutation of p53 leads to a cascade of protein deregulations, including the overexpression of the epidermal growth factor and the vascular endothelial growth factor, promoting tumor progression and angiogenesis.^8,9^ Mutant p53 is therefore a biomarker of cancer aggressiveness and chemo-resistance.^10^ As a result, gene therapies and chemotherapies targeting mutated p53 have been developed to enhance subefficient first line treatments.^11,12^

Detecting mutant p53 in tumor cells is of major clinical interest to orient patients to the most appropriate therapeutic strategies. However, diagnosis of mutant p53 is only performed by biopsy,^13^ a very invasive procedure that does not account for the heterogeneity of the tumor. Less invasive imaging approaches are also being developed using bioluminescence,^14^ fluorescence,^15^ or plasmonic imaging.^16^ However, these optical imaging techniques are limited to local or superficial studies, with numerous limitations for clinical applications. In contrast, positron emission tomography (PET) is a whole-body-scale and minimally invasive imaging technique with high sensitivity to quantitatively monitor physiological changes at the molecular level. Indirect imaging of p53 activity and downstream gene expression has already been performed with PET using [^124^I]­FIAU and a dual reporter gene,^17^ but this approach does not directly reflect the expression of mutant p53 itself. Yet, oligomerization of p53 occurs upon mutation, and the protein cannot be degraded, leading to its accumulation in the cytoplasm of tumor cells.^18^ This overexpression makes p53 a potential target for PET imaging, although radiotracers directly targeting this protein have never been designed. Developing PET tracers targeting the overexpression of p53 in tumor cells would represent a major advance, offering a valuable tool for noninvasive patient stratification, treatment monitoring, and supporting therapeutic development through companion diagnostics.

Among the different scaffolds described in the literature to target p53, compound CP31398 is a stryrylquinazoline derivative that stabilizes the conformation of the protein and induces cell death on various tumor cells.^19,20^ Although the binding mechanism of CP31398 to p53 is controversial,^21,22^ several reports describe the interaction of the molecule with the DNA-binding domain of the protein.^20,23^ Therefore, CP31398 appears to be a promising scaffold to design radiotracers for the PET imaging of p53. The reported toxicity of CP31398, which prevented further clinical development for this compound,^24^ is not an issue for an application in PET, as radiotracers are injected at trace doses (few micrograms).

CP31398 can be isotopically radiolabeled, *i.e*, without modification of the chemical structure and biological properties, by radiomethylation of the phenol moiety with carbon-11 (Figure 1). Isotopic labeling could also be possible on the dimethylamine function of the aliphatic chain, although possible regioselectivity issues can be anticipated with the secondary aromatic amine. Carbon-11 is a radioisotope of choice for isotopic labeling of drugs for PET imaging due to the omnipresence of carbon atoms in organic molecules. However, carbon-11 suffers from a short half-life (*t*_1/2_ = 20.4 min), which prevents the distribution of carbon-11 radiotracers to hospitals and limits its application to PET research centers. On the other hand, fluorine-18 (*t*_1/2_ = 109.8 min) is the most widespread radioisotope for PET imaging with ideal properties to consider further development to clinical applications. However, designing fluorine-18 labeled radiotracers of CP31398 requires modifications of the chemical structure of this compound, risking a loss of affinity to mutant p53. Because both radioisotopes display pros and cons, we decided to design both carbon-11 and fluorine-18 radiotracers based on the CP31398 scaffold to target mutant p53 with PET. Herein, we describe the synthesis of an original fluorinated derivative, namely FG-CP31398 (Figure 1), presenting a fluorinated glycol (FG) chain for aliphatic fluorination, a standard approach in radiochemistry with fluorine-18.^25^ We also describe the synthesis of its labeling precursor as well as the labeling precursor for isotopic labeling of CP31398 with carbon-11. Both radiosyntheses and quality controls of [^11^C]­CP31398 and [^18^F]­FG-CP31398 are presented, as well as the first *in vitro* evaluation of these radiotracers in both p53-transfected cells and tumor cells.

**Figure 1:**
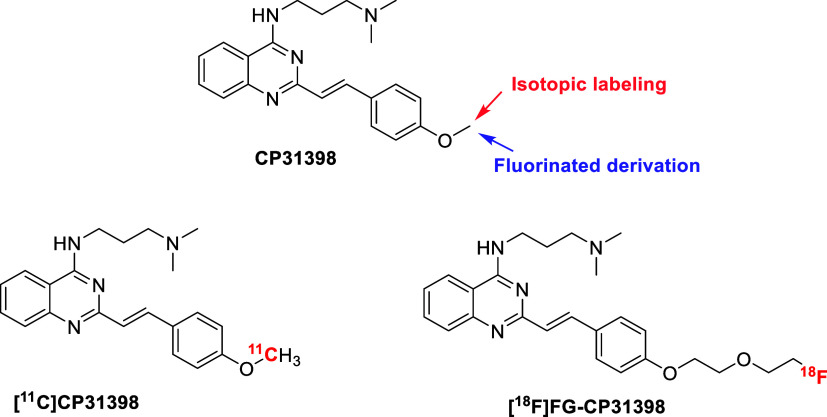
Structure of compound CP31398 with possible positions for isotopic labeling with carbon-11 or fluorinated derivation leading to two radiotracers presented herein, namely [^11^C]­CP31398 and [^18^F]­FG-CP31398.

In the course of this study, we also explored the synthesis and radiosynthesis of another fluorinated derivative, namely, F-CP31398, which bears a fluorine atom on position 7 of the quinazoline core, representing the minimal chemical modification possible from the CP31398 scaffold. Unfortunately, although we succeeded in synthesizing the compound and its labeling precursor, attempts at radiolabeling were unsuccessful. These data are presented in Supporting Information.

## Results and Discussion

### Chemistry

#### Synthesis of Compound **CP31398** and Its Carbon-11 Labeling Precursor

Inspired by previous work,^26,27^ compound **CP31398** was synthesized in three steps from commercially available 2-methylquinazolin-4­(*3H*)-one by condensation with *p*-anisaldehyde followed by a coupling reaction with *N* ^1^ *,N* ^1^-dimethylpropane-1,3-diamine in the presence of benzotriazol-1-yloxytris­(dimethylamino)­phosphonium hexafluorophosphate (BOP) and 1,8-diazabicyclo[5.4.0]­undec-7-ene (DBU) (Scheme 1). Overall, **CP31398** was synthesized as a chlorhydrate salt in an improved 69% yield compared to the synthesis of Sutherland et al. (44%).^26^ Aiming to perform isotopic labeling with carbon-11 by radiomethylation from a phenol precursor, demethylation of **CP31398** in the conditions of Newman et al.^28^ using sodium sulfide at high temperature in *N*-methyl-2-pyrrolidone (NMP) afforded the phenol precursor **2** in 75% yield (Scheme 1).

**Scheme 1:**
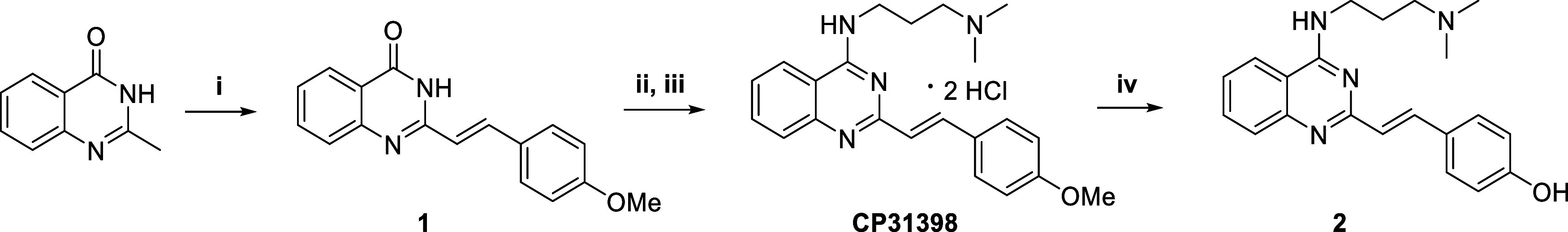
Synthesis of Compound **CP31398** and Its Labeling Precursor **2** ^a^

a Reagents and conditions: (i) *p*-anisaldehyde, AcOK, AcOH, 110 °C, 30h, 75%; (ii) *N* ^*1*^ *,N* ^*1*^-dimethylpropane-1,3-diamine, BOP, DBU, CH_3_CN, r.t., 24h; (iii) HCl, MeOH, r.t., 92% over two steps; (iv) Na_2_S, NMP, 140 °C, 6h, 75%.

#### Syntheses of Fluorinated Analogue **FG-CP31398** and Its Labeling Precursor

Compound **FG-CP31398** bears a fluorinated FG chain on the phenol moiety of the CP31398 scaffold and could be radiolabeled with fluorine-18 by type 2 nucleophilic substitution (S_N_2) from a tosylate precursor. Therefore, compound **FG-CP31398** and its labeling precursor **3** were both obtained in one step from previously synthesized phenol **2** by *O*-alkylation using either 2-(2-fluoroethoxy)­ethyl 4-methylbenzenesulfonate^29^ or commercially available diethylene glycol di­(*p*-toluenesulfonate) (Scheme 2). **FG-CP31398** and compound **3** were obtained in moderate yields of 38% and 25%, respectively, explained by the several purification steps necessary to obtain compounds with purities over 95%.

**Scheme 2:**
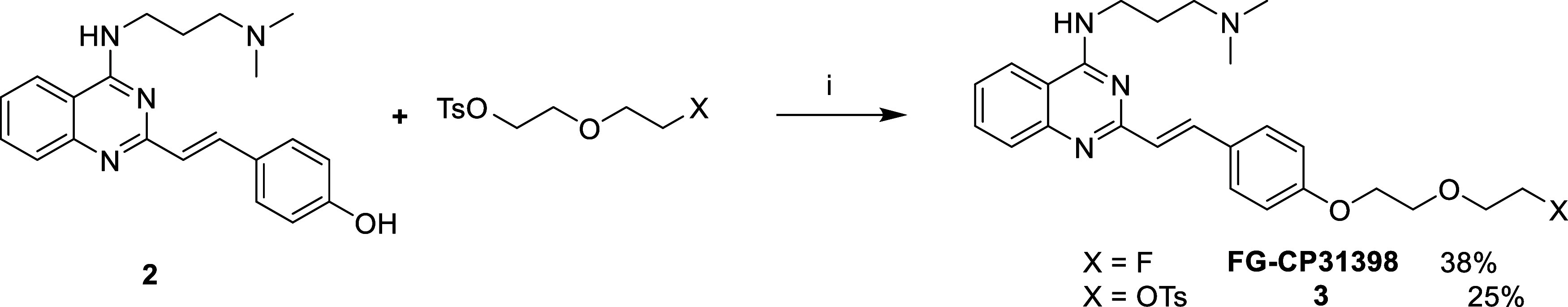
Synthesis of **FG-CP31398** and the Tosylate Precursor **3** in One Step from Phenol **2** by *O*-Alkylation^a^

a Reagents and conditions: (i) Cs_2_CO_3_, CH_3_CN, r.t. 24 h.

### Radiochemistry

#### Isotopic Radiolabeling of **CP31398** with Carbon-11

The radiosynthesis of compound [^11^C]**CP31398** can be performed by radiomethylation of phenol precursor **2** using either [^11^C]­CH_3_I or [^11^C]­CH_3_OTf as methylation agents under basic conditions (Scheme 3). [^11^C]­CH_3_I is prepared from cyclotron-produced [^11^C]­CO_2_ by the “gas method” described by Larsen et al.^30^ using a TRACERlab FX C Pro synthesizer, and [^11^C]­CH_3_OTf is obtained from [^11^C]­CH_3_I as reported by Jewett.^31^

**Scheme 3:**
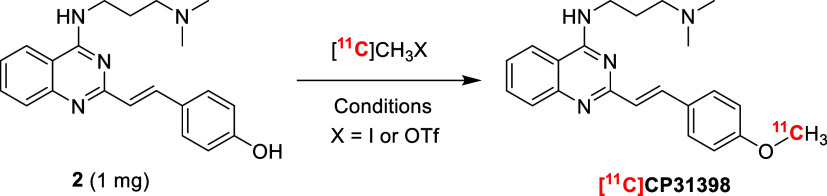
Radiomethylation of Phenol **2** Using Either [^11^C]­CH_3_I or [^11^C]­CH_3_OTf under Different Reaction Conditions to Afford Compound [^11^C]**CP31398**

Optimization of the radiomethylation conditions was performed with 1 mg of precursor **2** using the FX C Pro module, and the results are reported in Table 1. All reactions were carried out for 5 min, after which semipreparative high-performance liquid chromatography (HPLC) was performed on a reverse-phase column to isolate compound [^11^C]**CP31398**. A representative chromatogram is presented in the Supporting Information (Figure S1). The decay-corrected radiochemical yield (RCY) of the reaction was measured as the ratio of the isolated [^11^C]**CP31398** activity over the initial activity of [^11^C]­CH_3_I or [^11^C]­CH_3_OTf measured by the γ counter of the module reactor. A standard radiomethylation protocol using [^11^C]­CH_3_I and sodium hydroxide in acetone at 50 °C did not afford the desired radiotracer (Table 1, entry 1). Using the more reactive [^11^C]­CH_3_OTf methylation agent under the same conditions resulted in the same conclusion (Table 1, entry 2). Acetone was replaced by dimethylformamide (DMF) to improve the solubility of **2** and to reach a higher reaction temperature. In this context, traces of [^11^C]**CP31398** were observed using [^11^C]­CH_3_I and cesium carbonate as a base at 80 °C, together with the formation of radioactive side products (Table 1, entry 3). Using a base with a higher p*K*_a_, such as potassium *tert*-butanolate, resulted in the same conclusion (Table 1, entry 4). We hypothesized that the use of 2-butanone instead of DMF would reduce the formation of radioactive side products while ensuring good solubility of the precursor. Indeed, when the radiomethylation was performed in the presence of [^11^C]­CH_3_I and potassium *tert*-butanolate at 80 °C, traces of [^11^C]**CP31398** without any side products were observed (Table 1, entry 5). Using the more reactive [^11^C]­CH_3_OTf methylation agent under the same conditions afforded [^11^C]**CP31398** in 42% RCY without the formation of any side products (Table 1, entry 6). Finally, the whole radiochemical process was automated using the TRACERlab FX C Pro module and the optimized conditions. Semipreparative HPLC purification performed in a mixture of aqueous sodium acetate (0.5 M) and ethanol afforded ready-to-inject [^11^C]**CP31398** (0.6–1.6 GBq) in 40 ± 13% (*n* = 6) RCY within 40 min (nondecay corrected activity yield of 10 ± 3%). Quality control performed by reverse-phase analytical HPLC with ultraviolet (UV) and γ detection confirmed the identity of the product (see Figure S2 in Supporting Information). [^11^C]**CP31398** was obtained with over 99% chemical and radiochemical purities and 39 ± 12 GBq/μmol (*n* = 6) molar activity (MA).

**Table 1:** Optimization of the Radiomethylation of Phenol **2** ^a^

Entry^b^	methylation agent	base^c^	solvent	temperature (°C)	RCY^d^ (%)
**1**	[^11^C]CH_3_I	NaOH (3.0 equiv)	acetone	50	0
**2**	[^11^C]CH_3_OTf	NaOH (3.0 equiv)	acetone	50	0
**3**	[^11^C]CH_3_I	Cs_2_CO_3_ (3.0 equiv)	DMF	80	<1
**4**	[^11^C]CH_3_I	^*t*^BuOK (3.0 equiv)	DMF	80	<1
**5**	[^11^C]CH_3_I	^*t*^BuOK (3.0 equiv)	butanone	80	<1
**6**	[^11^C]CH_3_OTf	^*t*^BuOK (3.0 equiv)	butanone	80	42 ± 5

a Reactions were performed for 5 min on a TRACERlab FX C Pro module using 1 mg of precursor **2**.

b Experiments were performed in duplicate.

c The amount of base used is made precisely equivalent compared to the precursor (1 mg, 2.9 μmol).

d The radiochemical yield (RCY) is measured as the ratio of the [^11^C]**CP31398** activity isolated after semipreparative HPLC purification over the initial activity of [^11^C]­CH_3_I or [^11^C]­CH_3_OTf measured by the γ counter of the module reactor

#### Radiolabeling of **FG-CP31398** with Fluorine-18

Radiolabeling of **FG-CP31398** was performed by aliphatic S_N_2 from the tosylate precursor **3** in the presence of cyclotron-produced [^18^F]­fluoride, which was previously dried in the presence of potassium carbonate and Kryptofix-222 (K_222_) to form the K­[^18^F]­F- K_222_ complex (Scheme 4). The radiofluorination was performed with 4 mg of **3** in dimethyl sulfoxide (DMSO) to achieve complete solubility. Indeed, compound **3** was rather insoluble in acetonitrile, and a tentative of radiofluorination in this solvent at 100 °C did not yield any conversion.

**Scheme 4:**
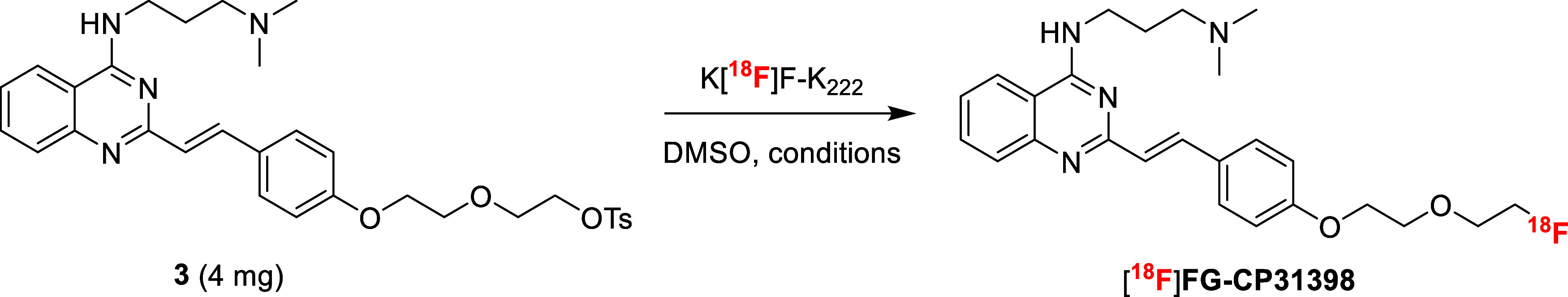
Radiofluorination of Precursor **3** by Aliphatic S_N_2 in the Presence of the K­[^18^F]­F–K_222_ Complex

Optimization of the reaction conditions was performed using a TRACERlab FX FN synthesizer and is depicted in Table 2. The radiochemical conversion (RCC) was measured by radioTLC. A representative radioTLC is presented in the Supporting Information (Figure S3). Upon reaction with K­[^18^F]­F/K_222_ at 80 °C for 5 min, the formation of [^18^F]**FG-CP31398** was observed on radioTLC with a conversion of 3% (Table 2, entry 1). No side product was observed, and the remaining activity was in the form of unreacted [^18^F]­F^–^. A longer reaction time (10 min) under the same conditions did not increase the conversion (Table 2, entry 2). In contrast, a higher temperature of 120 °C resulted in a double conversion of 6%, without any side products (Table 2, entry 3). Further increasing the temperature to 160 °C yielded a conversion of 37% into [^18^F]**FG-CP31398 (** Table 2, entry 4). Finally, a longer reaction time of 10 min at 160 °C did not result in a significant improvement of the conversion (Table 2, entry 5). As a result, the optimized conditions of radiofluorination were set at 160 °C for 5 min and the whole radiosynthesis process was automated on the Trasis AllinOne synthesizer. Semipreparative HPLC purification on a C18 column (see Figure S4 in the Supporting Information) followed by SPE formulation afforded the ready-to-inject radiotracer [^18^F]**FG-CP31398** (2.3 ± 0.5 GBq) in 10 ± 4% RCY (*n* = 4) within 45 min (nondecay corrected activity yield of 7.5 ± 2%). Quality control performed by reverse-phase analytical HPLC with UV and γ detection confirmed the identity of the product (see Figure S5 in the Supporting Information). [^18^F]**FG-CP31398** was obtained with 96% radiochemical puritiy and 80 ± 39 GBq/μmol (*n* = 4) MA.

**Table 2:** Optimization of the Radiofluorination of Precursor **3** ^a^

entry^b^	temperature (°C)	time (min)	RCC^c^ (%)
**1**	80	5	3 ± 1
**2**	80	10	3 ± 1
**3**	120	5	5 ± 1
**4**	**160**	**5**	**37 ± 4**
**5**	160	10	31 ± 6

a Reactions were performed in DMSO on a TRACERlab FX FN module using 4 mg of **3**.

b Experiments were performed in duplicate.

c The RCC was measured by radioTLC as the ratio of the area under the curve (AUC) of the [^18^F]**FG-CP31398** peak over the sum of the AUC of all peaks

### *In Vitro* Evaluation of Compounds [^11^C]­CP31398 and [^18^F]­FG-CP31398

#### Binding of [^18^F]**FG-CP31398** to p53

Compound **FG-CP31398** has been chemically modified compared to **CP31398**, which can lead to a modification of its affinity for p53. Therefore, binding experiments were performed on HEK-293T cells transiently transfected with a green fluorescent protein-fused version of wild-type p53 (GFP-p53wt) plasmid according to a procedure of the literature.^32^ Indeed, compound CP31398 is able to bind to both mutant and wild-type p53, so we assume that compound FG-CP31398 would behave the same way. Expression of p53 was verified by fluorescence microscopy (see Figure S6 in Supporting Information). To assess the binding potential of the molecule to its receptors, transfected cells (5 × 10^5^ cells per condition) were incubated with increasing concentrations of [^18^F]**FG-CP31398** (1–50 nM) with or without a large excess of CP31398 (100×). After several washing steps in cold phosphate-buffered saline (PBS), the cell pellet radioactivity was counted in a γ counter (Figures 2 and S7 in the Supporting Information). An increase in radioactivity was observed in the cell pellets together with the increase of [^18^F]**FG-CP31398** concentration, suggesting the binding of the radiotracer to the cells. The presaturation experiments with a 100-fold excess of the nonradioactive compound **CP31398** show a significant (*p* < 0.0005) decrease of *ca.* 80% of the radioactivity of the cell pellets upon incubation with 50 nM of [^18^F]**FG-CP31398** (Figure 2), indicating that the radiotracer binding is predominantly specific.

**Figure 2:**
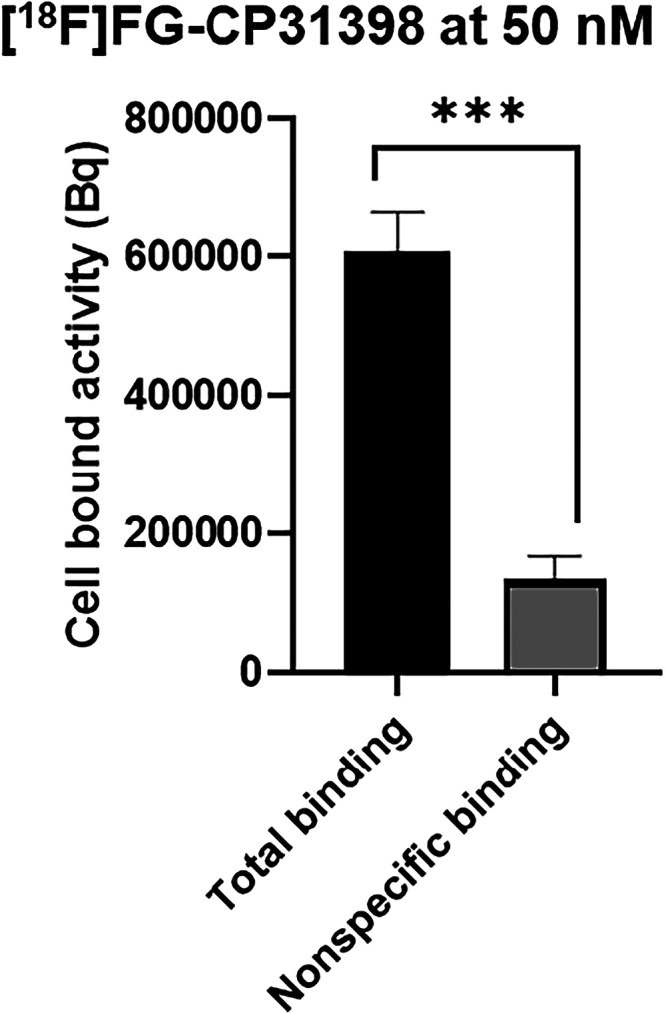
Binding experiments on HEK-293T cells transfected with the GFP-p53wt plasmid with an incubation of the cells at 50 nM of [^18^F]**FG-CP31398** with (gray) or without (black) presaturation with a 100-fold excess of **CP31398**.

#### Autoradiographies of [^11^C]**CP31398** and [^18^F]**FG-CP31398** on Tumor Slices

In order to verify if both radiotracers [^11^C]**CP31398** and [^18^F]**FG-CP31398** can bind to p53, the two radiotracers were incubated on different tumor sections expressing p53 to analyze the distribution of the radioactivity on the slices (autoradiography). Immunofluorescence screening of tumor slices from 6 different cell lines xenografted in mice (see Figure S8 in Supporting Information) using a p53 polyclonal antibody revealed that H358 lung cancer cells display the expression of p53 (Figure 3A). On the opposite, A549 lung cancer cells express very low p53 and will be used as a negative control. Tumors derived from H358 and A549 xenografts in nude mice were sectioned into 14 μm slices. These slices were then incubated for 2 h with either [^11^C]**CP31398** (164 ± 5 MBq) or [^18^F]**FG-CP31398** (124 ± 7 MBq) and were then washed twice in cold PBS. The remaining activity on the tumor slices was revealed by autoradiography (Figure 3B). A weak binding of [^18^F]**FG-CP31398** was observed on p53-expressing H358 cells, and the same result was observed for compound [^11^C]**CP31398**. These results tend to confirm that the mechanism of action of compound **CP31398** implies low binding to the protein, as depicted in few articles of the literature.^21^ Surprisingly, fixation of both radiotracers was observed in the p53-negative A549 cells. In addition, competition experiments performed on adjacent sections with nonradioactive compounds **CP31398** (for [^11^C]**CP31398**) or **FG-CP31398** (for [^18^F]**FG-CP31398**) showed a decrease of the signal in A549 cells, particularly significant for the [^11^C]**CP31398** (Figure 3B). This showcases a nonelucidated specific fixation mechanism. Overall, our findings indicate that neither CP31398 nor FG-CP31398 binds directly to p53 but instead interact with an unidentified target, nourishing the debate about the nonelucidated binding properties of CP31398.^21^ Although unsuitable for p53 imaging, the specific and unexpected binding patterns observed with [^11^C]­CP31398 and [^18^F]­FG-CP31398 indicate that these tracers could serve as mechanistic probes to identify alternative molecular targets of CP31398. Such studies may help resolve the long-standing debate of whether CP31398 acts through direct p53 stabilization or via indirect pathways such as DNA intercalation or interaction with other cellular proteins.

**Figure 3:**
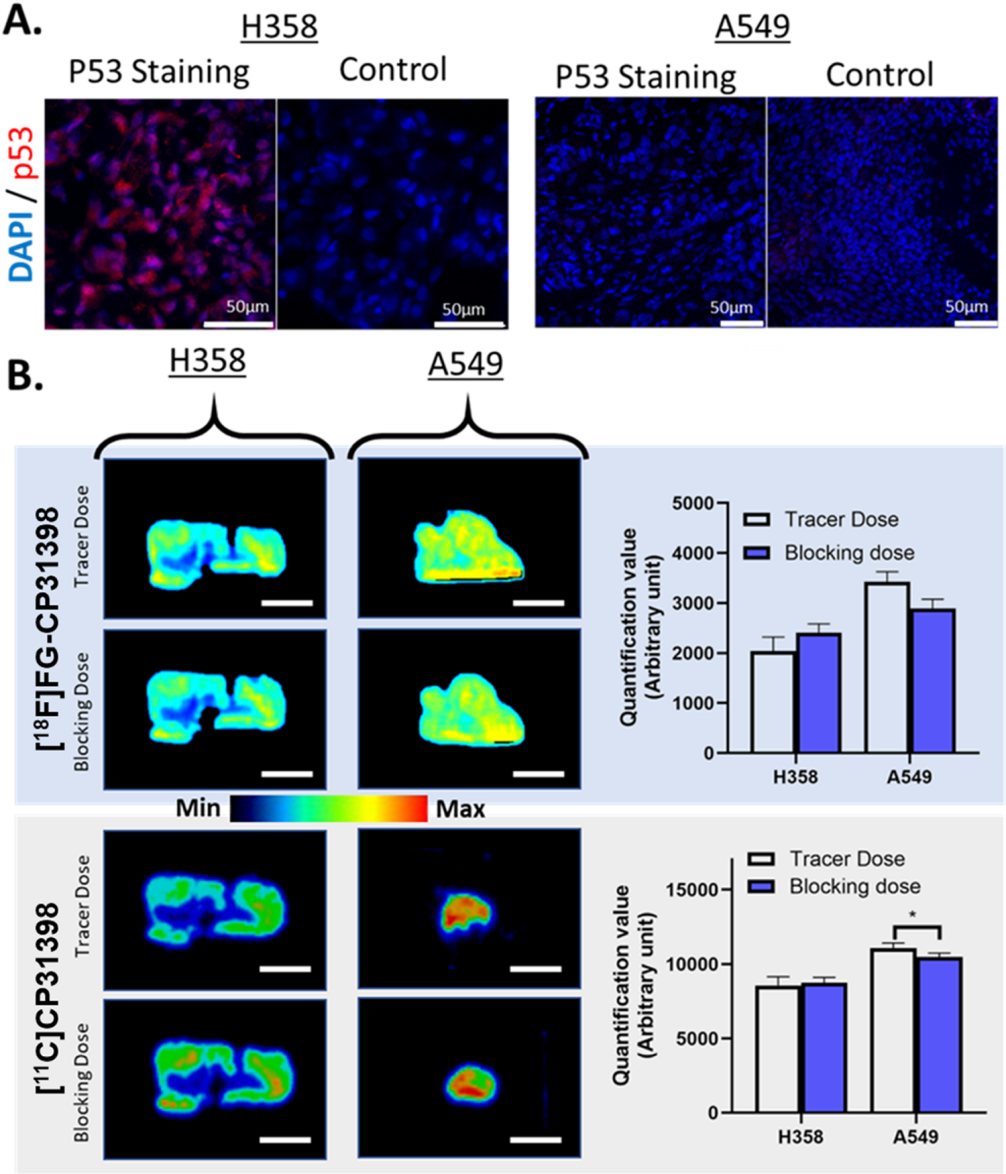
Comparative study of radioactive staining of [^18^F]**FG-CP31398** and [^11^C]**CP31398** on lung tumor slices. (A) Immunofluorescence of p53 and DAPI on H358 (p53^+^) and on A549 (p53^–^) tumor slices with the respective control (secondary antibody alone). (B) Autoradiography of H358 and A549 tumor slices after incubation with [^18^F]**FG-CP31398** (124 MBq) with and without presaturation with **FG-CP31398**; and with [^11^C]**CP31398** (164 MBq) with and without presaturation with **CP31398**. The white scale corresponds to 300 mm. Respective quantification of autoradiography slices (*n* = 4) allows determining the binding dose of the different radiotracers.

## Conclusion

In conclusion, aiming at developing original radiotracers for PET imaging of p53, we synthesized a fluorinated analogue of **CP31398** as well as its labeling precursor for radiofluorination with fluorine-18. We also synthesized a precursor for isotopic labeling of **CP31398** with carbon-11. Two radiotracers, namely [^11^C]**CP31398** and [^18^F]**FG-CP31398**, were successfully synthesized, and the automated process yielded ready-to-inject radiotracers meeting quality control criteria for *in vitro* and *in vivo* applications. Unfortunately, autoradiography experiments on tumor sections revealed that this family of radiotracers is able to show only weak binding to p53 and seems to show specific binding to one or several unknown targets, which remain to be determined. As a result, these radiotracers are not suitable for PET imaging of the mutant p53 protein. Nevertheless, these radiotracers could be used to elucidate the controversial mechanism of action of **CP31398**.

## Experimental Section

### Chemistry

Chemicals were purchased from chemical suppliers and used as received. Reactions were monitored by thin-layer chromatography (TLC) on aluminum precoated plates of silica gel 60F_254_ (VWR, France). The compounds were localized at 254 nm by using a UV-lamp. ^1^H and ^13^C NMR spectra were recorded on a Bruker Advance 400 MHz apparatus using DMSO-*d*_6_ as a solvent. The chemical shifts (δ) are reported in ppm (s, d, t, q, and b for singlet, doublet, triplet, quadruplet, and broad signal, respectively) and referenced with the solvent residual chemical shift. Coupling constants (*J*) are reported in Hertz (Hz). Melting points (mp) were measured with an Electrothermal IA9200 and are reported in °C. Liquid chromatography/mass spectroscopy analysis of synthesized compounds was realized on a Ultimate 3000 (Thermo Scientific) device equipped with an Acquity BEH 2.1 mm × 50 mm, 1.7 μm column (Waters). A gradient of water with 0.1% of formic acid and acetonitrile with 0.1% of formic acid (3% of CH_3_CN/HCHO for 2 min, then raising to 100% CH_3_CN/HCHO during 7 min, then decreasing to 3% during 1 min, and then keeping 3% for 2 min) at a flow rate of 0.3 mL/min was used. Analytical LCMS grade solvents were used for ultraperformance liquid chromatography/mass spectroscopy (LC/MS) analyses. Mass spectroscopy was performed with an LTQ Velos Pro Dual-Pressure linear Ion Trap mass spectrometer (Thermo Scientific) equipped with an electron spray ionization (ESI) chamber. Spectra were recorded at between 100 and 1000 *m*/*z*. High resolution mass spectrometry (HRMS) analysis was performed by the Small Molecule Mass Spectrometry platform of ICOA, (Orléans, France) by electrospray with positive (ESI+) ionization mode.

### *(E)-*2-(4-Methoxystyryl)­quinazolin-4­(*3H*)-one (**1**)

To a solution of 2-methylquinazolin-4­(3*H*)-one (800 mg, 1.0 equiv) in AcOH (15 mL) were added sodium acetate trihydrate (1.4 g, 2.0 equiv) and 4-methoxybenzaldehyde (1.2 mL, 2.0 equiv). The mixture was stirred for 24 h at 120 °C. After cooling to room temperature, ice–water (20 mL) was added, and the precipitate formed was filtered and washed with ice–water (10 mL) to afford **1** (1.0 g, 75%) as a brown powder. ^1^H NMR (DMSO*-d*_6_, 400 MHz): δ 12.29 (s, 1H), 8.09 (dd, *J =* 7.6 Hz, *J =* 1.2 Hz, 1H), 7.90 (d, *J =* 16.0 Hz, 1H), 7.79 (td, *J =* 7.1 Hz, *J =* 1.6 Hz, 1H), 7.63 (m, 3H), 7.45 (td, *J =* 7.1 Hz, *J =* 1.2 Hz, 1H), 7.03 (d, *J =* 8.7 Hz, 2H), 6.87 (d, *J =* 16.1 Hz, 1H), 3.82 (s, 3H) ppm. ^13^C NMR (DMSO*-d*_6_, 100 MHz): δ 162.1, 160.5, 152.1, 149.1, 137.9, 134.3, 129.2 (2C), 127.7, 126.8, 125.8, 125.7, 121.0, 118.8, 114.5 (2C), 55.3 ppm. Mp 295–298 °C. LC/MS: t_R_ = 7.26 min.; 533.57 *m*/*z* [M + H]^+^; 555.54 *m*/*z* [M + Na]^+^.

### *(E)-N* ^1^-(2-(4-Methoxystyryl)­quinazolin-4-yl)-*N* ^3^ *,N* ^3^-dimethylpropane-1,3-diamine Dihydrochloride (**CP31398**)

To a solution of **1** (600 mg, 1.0 equiv) in CH_3_CN (16 mL) were added BOP (1.90 g, 2.0 equiv) and DBU (653 μL, 2.0 equiv). The reaction mixture was stirred for 10 min under argon at room temperature. Then, 3-(dimethylamino)-1-propylamine (690 μL, 2.0 equiv) was added, and the solution was stirred for 24 h at room temperature under argon. The mixture was concentrated under vacuum, and the residue was dissolved in CH_2_Cl_2_ (20 mL). The organic phase was washed with water (2 mL × 20 mL) and brine (20 mL), dried over sodium sulfate, filtered, and concentrated under vacuum. HCl (4 M in MeOH, 6 mL) was added, followed by EtOAc (5 mL). The precipitate formed was filtered and washed with CH_3_CN (10 mL) to afford the dihydrochlorate salt of **CP31398** (840 mg, 92%) as a yellow solid. ^1^H NMR (DMSO*-d*_6_, 400 MHz): δ 14.75 (b, 1H), 10.51 (b, 1H), 10.31 (b, 1H), 8.60 (d, *J =* 8.6 Hz, 1H), 8.34 (d, *J =* 15.9 Hz, 1H), 7.99 (m, 1H), 7.88 (d, *J* = 8.6 Hz, 1H), 7.79 (d, *J =* 8.8 Hz, 2H), 7.70 (m, 1H), 7.18 (d, *J =* 15.9 Hz, 1H), 7.08 (d, *J* = 8.8 Hz, 2H), 3.94 (q, *J* = 6.0 Hz, 2H), 3.84 (s, 3H), 3.23 (m, 2H), 2.77 (s, 3H), 2.75 (s, 3H), 2.17 (m, 2H) ppm. LC/MS: t_R_ = 3.26 min.; 363.26 *m*/*z* [M + H]^+^. (see Figure S9 in Supporting Information for ^1^H NMR analysis). Data in accordance with literature.^26^

### *(E)-*4-(2-(4-((3-(Dimethylamino)­propyl)­amino)­quinazolin-2-yl)­vinyl)­phenol (**2**)

To a solution of **CP31398** dihydrochloride (500 mg, 1.0 equiv) in NMP (15 mL) was added Na_2_S (860 mg, 10.0 equiv), and the mixture was stirred for 6h at 140 °C. The reaction mixture was then quenched with water (20 mL), and the aqueous layer was extracted with CH_2_Cl_2_ (3 mL × 20 mL). The combined organic layers were washed with brine (20 mL), dried over sodium sulfate, filtered, and concentrated under vacuum to afford **2** (300 mg, 75%) as a yellow solid. ^1^H NMR (DMSO*-d*_6_, 400 MHz): δ 8.27 (b, 1H), 8.15 (d, *J =* 8.8 Hz, 1H), 7.83 (d, *J =* 16.0 Hz, 1H), 7.71 (m, 1H), 7.64 (d, *J =* 8.8 Hz, 1H), 7.24 (m, 1H), 7.15 (d, *J =* 8.8 Hz, 2H), 7.42 (m, 1H), 6.91 (d, *J =* 16 Hz, 1H), 6.81 (d, *J =* 8.8 Hz, 2H), 3.66 (m, 2H), 2.66 (m, 2H), 2.39 (s, 6H), 1.92 (t, *J =* 9.0 Hz, 2H) ppm. ^13^C NMR (DMSO*-d*_6_, 100 MHz): δ 173.7, 160.4, 159.0, 158.2, 149.9, 136.1, 132.3, 128.9 (2C), 127.3, 126.1, 124.7, 122.5, 115.7 (2C), 113.8, 57.1, 48.4, 45.1 (2C), 26.3 ppm. Mp 185–191 °C. HR-ESI­(+)-MS *m*/*z* calcd for C_21_H_25_N_4_O: 349.2023; found 349.2028 [M + H]^+^ (see Figures S10–S12 in Supporting Information for NMR and HRMS analysis).

### *(E)-N* ^1^-(2-(4-(2-(2-Fluoroethoxy)­ethoxy)­styryl)­quinazolin-4-yl)-*N* ^3^ *,N* ^3^-dimethylpropane-1,3-diamine (**FG-CP31398**)

To a solution of **2** (30 mg, 1.0 equiv) in CH_3_CN (3 mL) were added Cs_2_CO_3_ (60 mg, 2.0 equiv) and 2-(2-fluoroethoxy)­ethyl 4-methylbenzenesulfonate^29^ (47 mg, 2.0 equiv). The solution was stirred for 24h at room temperature, under argon. The reaction mixture was then concentrated to dryness, and the residue was dissolved in CH_2_Cl_2_ (5 mL). The organic phase was washed with water (2 mL × 10 mL) and brine (10 mL), dried over sodium sulfate, filtered, and concentrated under vacuum. The residue was purified by chromatography using CH_2_Cl_2_/MeOH 9/1 v/v as eluent to afford compound **FG-CP31398** (14 mg, 38%) as a yellow oil. ^1^H NMR (DMSO*-d*_6_, 400 MHz): δ 8.25 (t, *J =* 5.6 Hz, 1H), 8.14 (d, *J =* 8.2 Hz, 1H), 7.86 (d, *J =* 16.0 Hz, 1H), 7.70 (m, 1H), 7.63 (m, 3H), 7.42 (m, 1H), 6.99 (m, 3H), 4.55 (td, *J =* 48.0 Hz, *J =* 4.1 Hz, 2H), 4.15 (m, 2H), 3.72 (m, 6H), 2.39 (t, *J =* 7.3 Hz, 2H), 2.20 (s, 6H), 1.85 (q, *J =* 7.4 Hz, 2H) ppm. ^13^C NMR (DMSO*-d*_6_, 100 MHz): δ 160.1, 158.9, 158.8, 149.8, 135.4 (2C), 132.2, 128.7 (2C), 127.2, 127.0, 124.7, 122.4, 114.7 (2C), 113.7, 82.9 (d, *J* = 165 Hz), 69.6 (d, *J* = 19 Hz), 68.8, 67.1, 57.0, 45.0 (2C), 39.1, 26.3 ppm. HR-ESI­(+)-MS *m*/*z* calcd for C_25_H_32_FN_4_O_2_: 439.2509; found 439.2505 [M + H]^+^ (see Figures S13–S15 in Supporting Information for NMR and HRMS analysis).

### *(E)-*2-(2-(4-(2-(4-((3-(Dimethylamino)­propyl)­amino)­quinazolin-2-yl)­vinyl)­phenoxy)­ethoxy)­ethyl 4-Methylbenzenesulfonate (**3**)

To a solution of **2** (55 mg, 1.0 equiv) in CH_3_CN (3 mL) were added Cs_2_CO_3_ (104 mg, 2.0 equiv) and diethylene glycol di­(*p*-toluenesulfonate) (133 mg, 2.0 equiv). The resulting solution was stirred for 24h at room temperature, under argon. The reaction mixture was then concentrated to dryness, and the residue was dissolved in CH_2_Cl_2_ (5 mL). The organic phase was washed with water (2 mL × 15 mL) and brine (15 mL), dried over sodium sulfate, filtered, and concentrated under vacuum. The residue was purified by chromatography using CH_2_Cl_2_/MeOH/NH_4_OH 95/5/0.1 v/v/v as the eluent to afford **3** (25 mg, 25%) as a yellow oil. ^1^H NMR (DMSO*-d*_6_, 400 MHz): δ 8.27 (t, *J* = 2.2 Hz, 1H), 8.14 (d, *J =* 8.2 Hz, 1H), 7.87 (d, *J =* 15.7 Hz, 1H), 7.79 (d, *J* = 8.4 Hz, 2H), 7.72 (m, 1H), 7.65 (m, 3H), 7.44 (m, 3H), 6.99 (m, 3H), 4.16 (m, 2H), 4.07 (m, 2H), 3.66 (m, 6H), 2.39 (s, 3H), 2.29 (m, 6H), 1.88 (m, 2H) ppm. ^13^C NMR (DMSO*-d*_6_, 100 MHz): δ 159.2, 149.9, 145.7, 137.6, 135.7, 132.6, 130.1, 128.9 (2C), 128.0 (2C), 127.6, 125.5 (2C), 122.7, 114.8 (2C), 113.8, 70.0, 68.8, 68.7, 68.0, 66.8, 64.0, 62.5, 62.3, 54.9, 50.9 (2C), 37.5, 21.1, 20.8 ppm. HR-ESI­(+)-MS *m*/*z* calcd for C_32_H_39_N_4_O_5_S: 591.2641; found 591.2626 [M + H]^+^ (see Figures S16–S18 in Supporting Information for NMR and HRMS analysis).

### Radiochemistry

#### General Procedure for Quality Control

Quality control was realized by analytical HPLC performed using a 717plus Autosampler system, a 1525 binary pump, a 2996 photodiode array detector (Waters), and a Flowstar LB 513 (Berthold, France) γ detector. The system was operated with Empower 3 (Waters) software. HPLC was realized on a reverse-phase analytical Symmetry C18 (150 mm × 3.9 mm, 5 μm, Waters) column using a mixture of H_2_O/CH_3_CN/PicB7 as eluent. The chemical identification of the peak was assessed by comparing the retention time of the radiotracer with the retention time of the nonradioactive reference (*t*_Rref_). For acceptance, the retention time must be within the *t*_Rref_ ± 10% range. Radiochemical and chemical purities were calculated as the ratio of the AUC of the radiotracer peak over the sum of the AUC of all other peaks on γ and UV chromatograms, respectively. Molar activity (MA) was calculated as the ratio of the activity of the collected peak of the radiotracer measured in an ionization chamber (Capintec, Berthold, France) over the molar quantity of the nonradioactive compound determined using a calibration curve. MA is calculated as the mean value of three consecutive runs.

#### Radio-Thin Layer Chromatography

Radio-thin Layer Chromatography (radioTLC) was performed on precoated plates of silica gel 60F254 (Merck) and eluted with ethyl acetate or a mixture of MeOH/CH_2_Cl_2_. Radioactive compounds were detected using a MiniScan and Flow-Count radioactive detection system (Bioscan, France) operated with Chromeleon software (Thermo Scientific).

#### Radiolabeling of [^11^C]**CP31398** with Carbon-11

All reactions were performed using the TRACERlab FX C Pro automated module. No carrier-added [^11^C]­CO_2_ (25–50 GBq) was produced via the ^14^N­(p, α)^11^C nuclear reaction by irradiation of a [^14^N]­N_2_ target containing 0.15–0.5% of the aqueous O_2_ on a cyclone 18/9 cyclotron (18 MeV, IBA, Belgium). [^11^C]­CO_2_ was subsequently reduced to [^11^C]­CH_4_ by hydrogenation on Shimalite nickel and iodinated to [^11^C]­CH_3_I at 750 °C in the presence of I_2(g)_ following the recirculating process described by Larsen et al.^30^ [^11^C]­CH_3_I was converted into [^11^C]­CH_3_OTf by reaction over AgOTf at 200 °C according to the method of Jewett.^31^ [^11^C]­CH_3_OTf was bubbled into a solution of compound **2** (1 mg, 2.9 μmol) and potassium *tert*-butanolate (1 mg, 8.9 μmol) in 2-butanone (300 μL) at −20 °C for 3 min. At this time, the initial activity value of [^11^C]­CH_3_OTf was measured by means of a calibrated detector of the module. The mixture was heated at 85 °C for 5 min. Upon cooling to 60 °C, the crude product was quenched with a mixture of aqueous sodium acetate (0.5 M)/ethanol (60/40 v/v, 1 mL). The crude was purified by reverse-phase semipreparative HPLC (Waters Symmetry C18 7.8 mm × 300 mm, 7 μm) with a 515 HPLC Pump (Waters) using a mixture of aqueous sodium acetate (0.5 M)/ethanol (60/40 v/v, 5 mL/min) as eluent. UV detection (K2501, Knauer, Germany) was performed at 254 nm. Ready-to-inject [^11^C]­CP31398 (4–6 GBq) was obtained in 40 ± 13% RCY within 40 min (*n* = 6). The RCY is decay-corrected and was calculated as the ratio of the final activity of [^11^C]**CP31398** measured in an activity chamber (Capintec, Berthold, France) over the initial activity of [^11^C]­CH_3_OTf measured in the module reactor by means of a calibrated γ detector.

Quality control of [^11^C]**CP31398** was performed according to the general procedure using a mixture of H_2_O/CH_3_CN (7/3 v/v) as the eluent and UV detection at 254 nm. [^11^C]**CP31398** was obtained with a chemical and a radiochemical purity >99% and an average specific activity of 39 ± 12 GBq/μmol (*n* = 6).

#### Radiolabeling of [^18^F]**FG-CP31398** with Fluorine-18

Reactions were carried out using a TRACERlab FX FN module (GE Healthcare, Sweden) or an AllInOne (Trasis, Belgium) synthesizer. No carrier-added [^18^F]­fluoride ion (20–30 GBq) was produced via the ^18^O­(p,n)^18^F nuclear reaction by irradiation of a 2 mL [^18^O]­water (>97% enriched, Rotem, Israël) target with an IBA Cyclone-18/9 (IBA, Belgium) cyclotron. [^18^F]­F^–^ was trapped on an ion-exchange resin QMA light (Waters) and eluted in the reactor using a solution of 4,7,13,16,21,24-hexaoxa-1,10-diazabicyclo[8.8.8]­hexacosane (K_222_, 12–15 mg, 31.9–40.0 μmol) and potassium carbonate (2 mg, 14.8 μmol) in a mixture of CH_3_CN (800 μL) and H_2_O (200 μL). The resulting complex was dried upon heating at 60 °C for 7 min under vacuum and a stream of helium, followed by heating at 120 °C for 5 min under vacuum only. A solution of **3** (4 mg, 6.8 μmol) in DMSO (700 μL) was added, and the mixture was heated at 160 °C for 5 min. Upon cooling to room temperature, the mixture was diluted with a mixture of H_2_O/CH_3_CN/TFA (85/15/0.1 v/v/v, 4 mL), and the crude product was passed through an Alumina N cartridge (Waters). Purification was realized by reverse-phase semipreparative HPLC (Waters Symmetry C18 7.8 mm × 300 mm, 7 μm) with a 515 HPLC Pump (Waters) using a mixture of H_2_O/CH_3_CN/TFA (85/15/0.1 v/v/v, 5 mL/min) as eluent. UV detection (K2501, Knauer, Germany) was performed at 254 nm. The purified compound was diluted with water (20 mL) and passed through a Sep-Pak C18 cartridge (Waters). The cartridge was rinsed with water (10 mL) and eluted with ethanol (2 mL), and the final compound was diluted with saline (0.9% w/v, 8 mL). Ready-to-inject [^18^F]­FG-CP31398 (1.2–2.0 GBq) was obtained in 10% ± 4 RCY (*n* = 4) within 60 min.

Quality control of [^18^F]**FG-CP31398** was performed according to the general procedure using a mixture of H_2_O/CH_3_CN (7/3 v/v) as the eluent and UV detection at 254 nm. [^18^F]**FG-CP31398** was obtained with a radiochemical purity of 96$ and an average specific activity of 80 ± 39 GBq/μmol (*n* = 4).

### *In Vitro* Evaluation of the Radiotracers

#### Cell Culture

The human cell line HEK-293T was purchased from the American Type Culture Collection. The cells were cultured in DMEM/F12 medium (Gibco, France) supplemented with 10% fetal bovine serum (Gibco, France), at 37 °C in a humidified atmosphere with 5% CO_2_.

#### Transfection with the GFP-p53 Wild-Type Plasmid^32^

10^6^ cells are seeded per 10 cm diameter Petri dish. After 24 h, the cells were transfected with 30 μG of a GFP-p53 plasmid (Addgene, #12091) and 60 μL of Lipofectamine 2000 transfection reagent (Thermo Fisher, France) according to the manufacturer’s instructions.

#### Fluorescence Microscopy

Frozen fixed tumor sections (14 μm) obtained from the laboratory tumor biobank were incubated with the primary rabbit antihuman p53 antibody (1:1000, Thermo Fisher, #PA5–27822). The following tumors were tested: A431, A549, CT26, H358, H1975, and U87. The slides were then incubated with the secondary antibody (Sigma-Aldrich #A0545) diluted 1:10,000 for 1 h at room temperature. Nuclei were counterstained with DAPI included in the mounting medium. For each immunofluorescence staining, a control slice is acquired with only the secondary antibody. Images of stained tumor sections were acquired using a Zeiss AxioCam fluorescence microscope (Carl Zeiss, Germany).

#### Binding Experiments

Transfected HEK-293T cells (5 × 10^5^ cells) were exposed to [^18^F]**FG-CP31398**, with or without 100× molar excess of nonradiolabeled **FG-CP31398** ligand for 1.5 h at 37 °C under agitation. Three molar concentrations of [^18^F]**FG-CP31398** were tested: 1, 10, and 50 nM. After multiple washes, the cell-bounded activity was measured using a Wizard2 γ counter (PerkinElmer; France). Statistical analyses were conducted using GraphPad Prism (v10.0.1). A two-tailed Student’s *t* test was applied, and results were deemed statistically significant for *p*-values <0.05.

#### Autoradiography

Tumor sections were incubated either with [^18^F]**FG-CP31398** (124 ± 7 MBq) or [^11^C]**CP31398** (164 ± 5 MBq) according to a previously described protocol.^33^ Specific binding was assessed using an excess of unlabeled **FG-CP31398** (4 μmol) or **CP31398** (3 μmol), respectively. Sections were incubated for 20 min in Tris Buffer (50 mM) with NaCl (120 mM) adjusted to pH 7.4. The unbound excess ligands were removed by two 5 min wash cycles in cold buffer and then a final rinse in cold deionized water. Slides were placed in contact with a phosphor screen in a cassette (Molecular Dynamics) overnight. Images were acquired at 50 μm resolution with an imager (Storm 860 Molecular Imager, Molecular Dynamics).

## Supplementary Material



## Notes

Word document (docx) containing: HPLC analysis of [^11^C]­CP31398; TLC and HPLC analysis of [^18^F]­FG-CP31398; Microscopy of HEK 293T transfected cells; Immunofluorescence of tumor sections; ^1^H, ^13^C NMR and HRMS analysis (PDF)
