# Supplementary material for: Carbon-11 Isotopic Radiolabeling of CP31398 and Development of a Fluorine-18 Derivative to Target Protein p53 with PET Imaging
Source: ACS Omega. 2026 Jan 28;11(5):7142–50. doi: 10.1021/acsomega.5c06415 (PMC12902872; doi:10.1021/acsomega.5c06415)
Supplement: Supplementary file 1 [file ao5c06415_si_001.pdf]

Supporting information

Carbon-11 isotopic radiolabeling of CP31398 and development of a fluorine-18 derivative to target protein p53 with PET imaging

Sébastien Beuché[1], Soizic Martin Aubert[1], Philippe Robin[2], Caroline Denis[1], Denis Servent[2], Bertrand Kuhnast[1], Charles Truillet[1] and Fabien Caillé[1,*]

[1]Université Paris-Saclay, Inserm, CNRS, CEA, Laboratoire d'Imagerie Biomédicale Multimodale Paris-Saclay (BioMaps), 4 place du Général Leclerc 91401 Orsay, France
[2]Université Paris-Saclay, CEA, Département Médicaments et Technologies pour la Santé (DMTS), SIMoS, 91191 Gif sur Yvette, France

Contents

HPLC analysis of [^{11}C]**CP31398**……………………………………………………………p.2
TLC and HPLC analysis of [^{18}F]**FG-CP31398**…………………………………………p.3
Microscopy of HEK 293-T tranfected cells……………………………………………………p.5
Immunofluorescence of tumor sections…………………………………………………..p.6
^{1}H, ^{13}C-NMR and HRMS analysis……………………………………………………p.7
Synthesis of compound F-CP31398 and labeling precursor………………………...p.12
Radiosynthesis attemps for [^{18}F]F-CP31398……………………………………..p.13
Material and methods for F-CP31398 and labeling precursor………………………p.14
^{1}H, ^{13}C-NMR and HRMS analysis for F-CP31398 and labeling precursor…………p.17
References……………………………………………………………………………..p.19

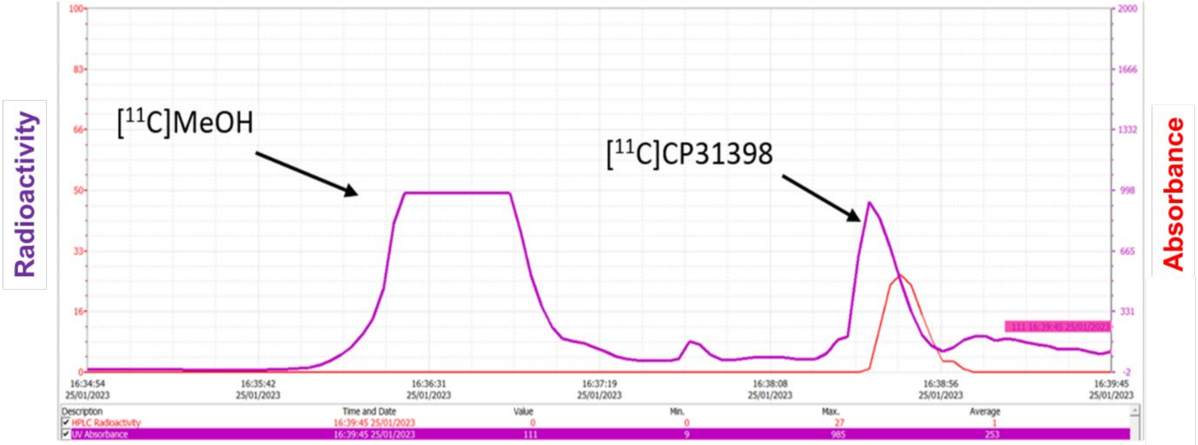

Figure S1. HPLC purification of [11C]**CP31398** realized by semi-preparative HPLC on a reverse phase C18 Symmetry column (7.8 x 300 mm, 7 µm) using sodium acetate (0.5 M)/ethanol (60/40 v/v) as mobile phase at 5 mL/min. Gamma chromatogram is presented in violet and UV (254 nm) chromatogram is presented in red.

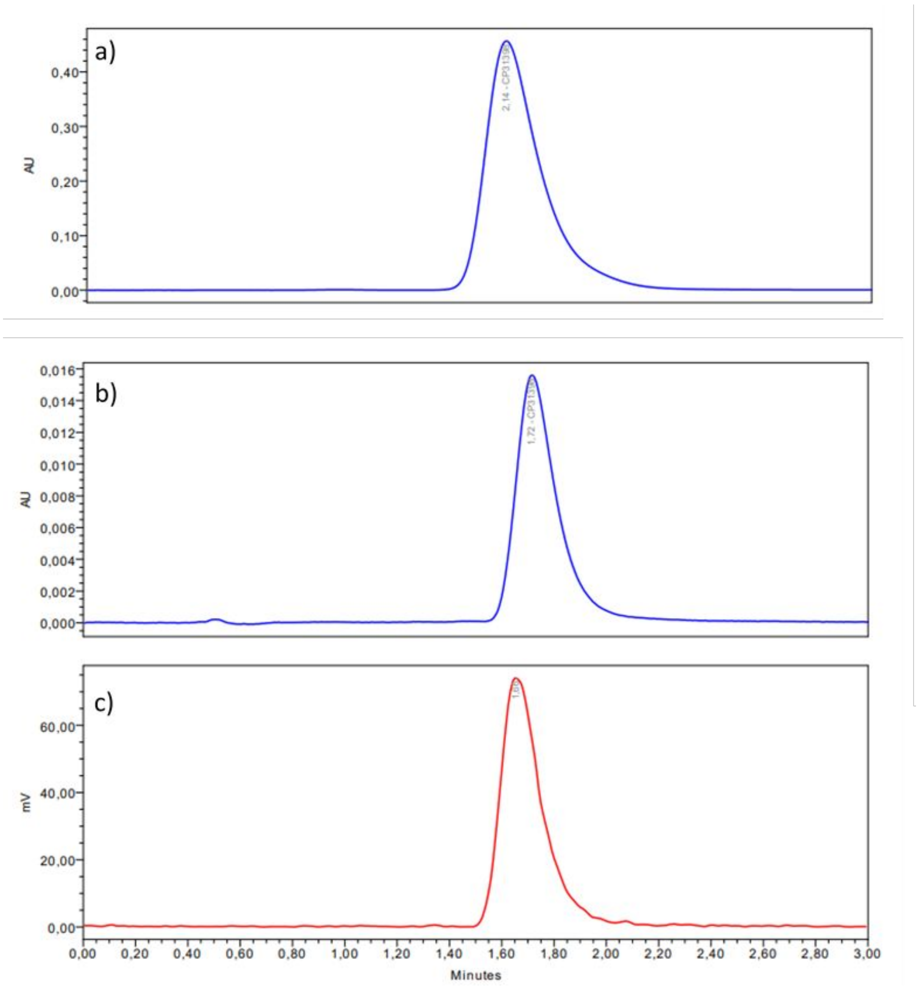

Figure S2. Quality control of [11C]**CP31398** realized by analytical HPLC on a reverse phase C18 Symmetry column (150 x 3.9 mm, 5 µm) using H_2O/CH_3CN (7/3 v/v) as mobile phase at 2 mL/min. a) UV chromatogram of the reference compound **CP31398** recorded at 359 nm. b) UV chromatogram of [11C]**CP31398** recorded at 359 nm; c) Gamma chromatogram of [11C]**CP31398**.

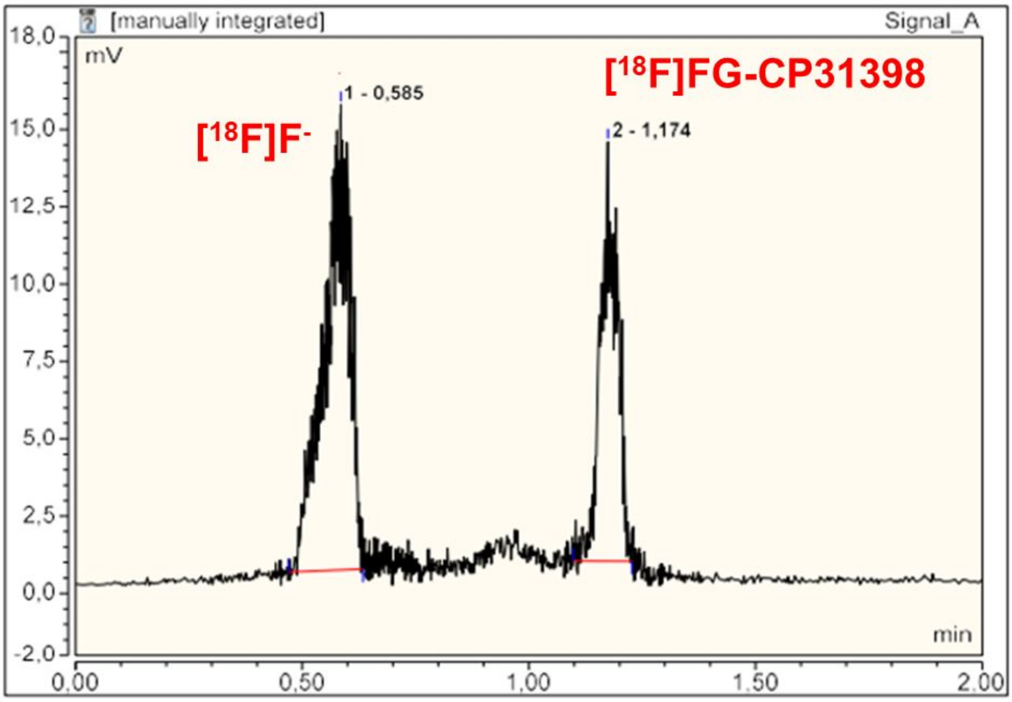

Figure S3. RadioTLC analysis of the crude reaction of fluorination of precursor **3** with [18F]F^{-} in DMSO at 160 °C for 5 min, leading to a 37% RCC into [18F]**FG-CP31398**.

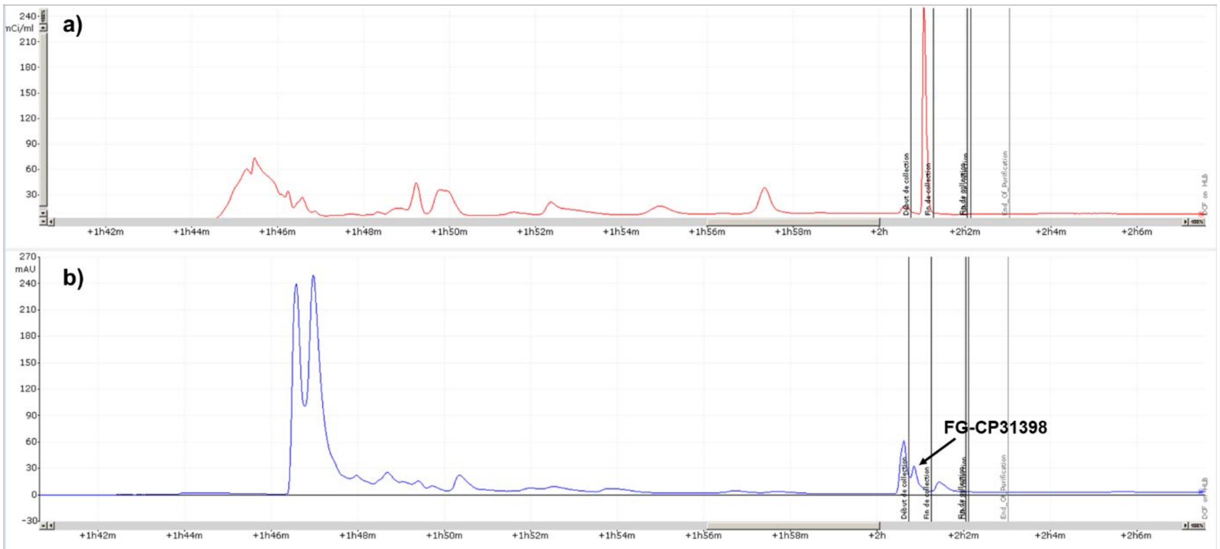

Figure S4. HPLC purification of [18F]**FG-CP31398** performed by semi-preparative HPLC on a reverse phase C18 Symmetry column (7.8 x 300 mm, 7 µm) using $H_2O/CH_3CN/TFA$ 85/15/0.1 v/v/v as mobile phase at 5 mL/min. (A) Gamma chromatogram; (B) UV chromatogram recorded at 254 nm.

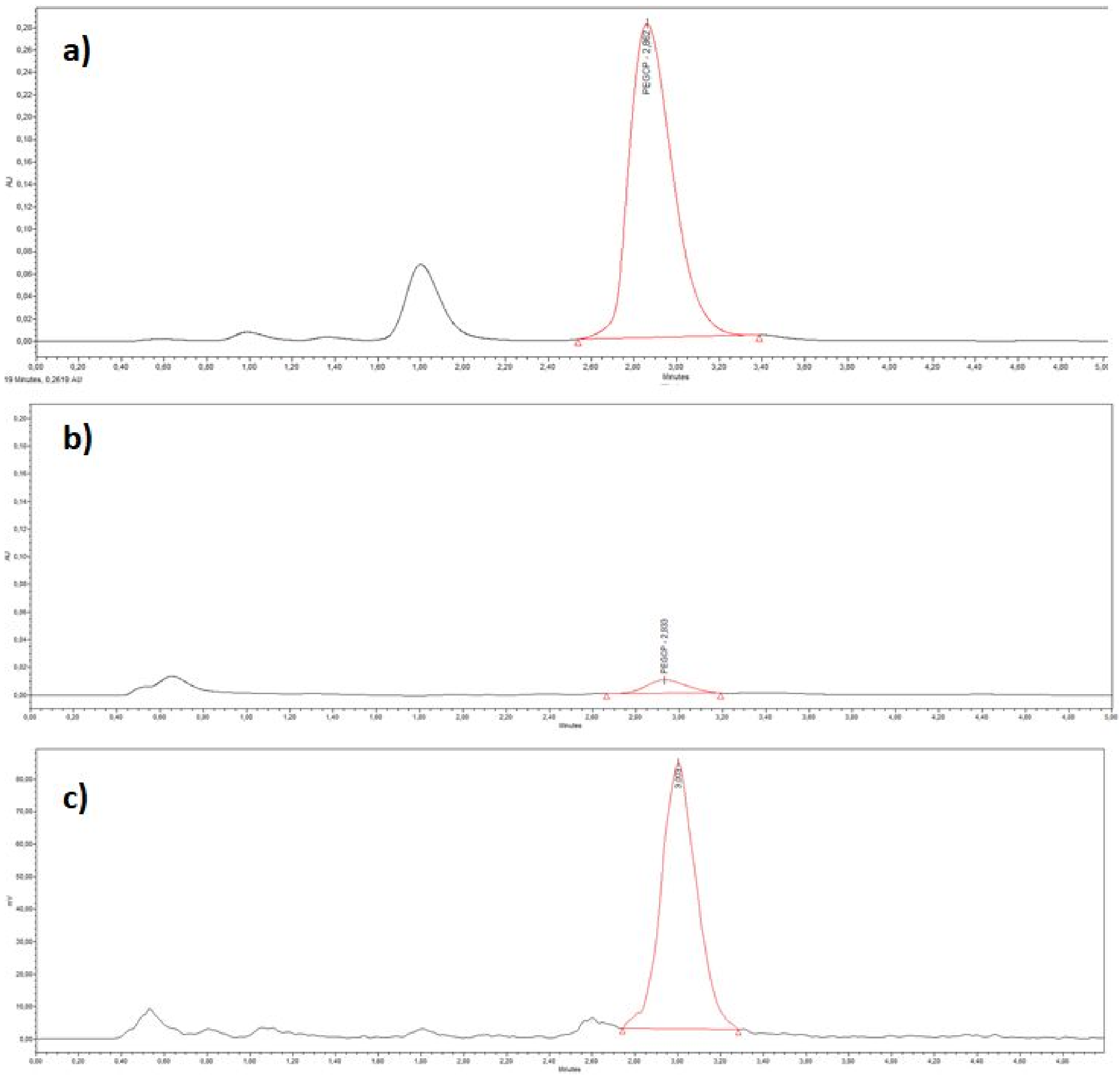

Figure S5. Quality control of [^{18}F]**FG-CP31398** realized by analytical HPLC on a reverse phase C18 Symmetry column (150 x 3.9 mm, 5 µm) using H_2O/CH_3CN (7/3 v/v) as mobile phase at 2 mL/min. a) UV chromatogram of the reference compound **FG-CP31398** recorded at 359 nm. b) UV chromatogram of [^{18}F]**FG-CP31398** recorded at 359 nm; c) Gamma chromatogram of [^{18}F]**FG-CP31398**.

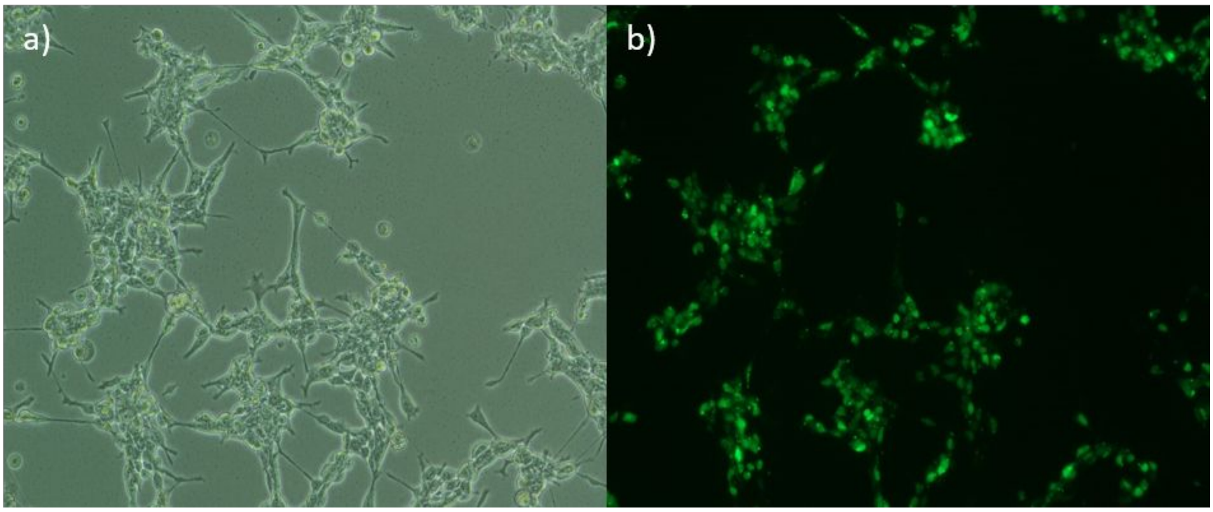

Figure S6. Microscopy of HEK-293T cells transfected with the GFP-p53wt plasmid observed with a Nikon Eclipse TI (x10). a) Direct observation of the cells; b) Fluorescence (GFP/FITC filter set) microscopy.

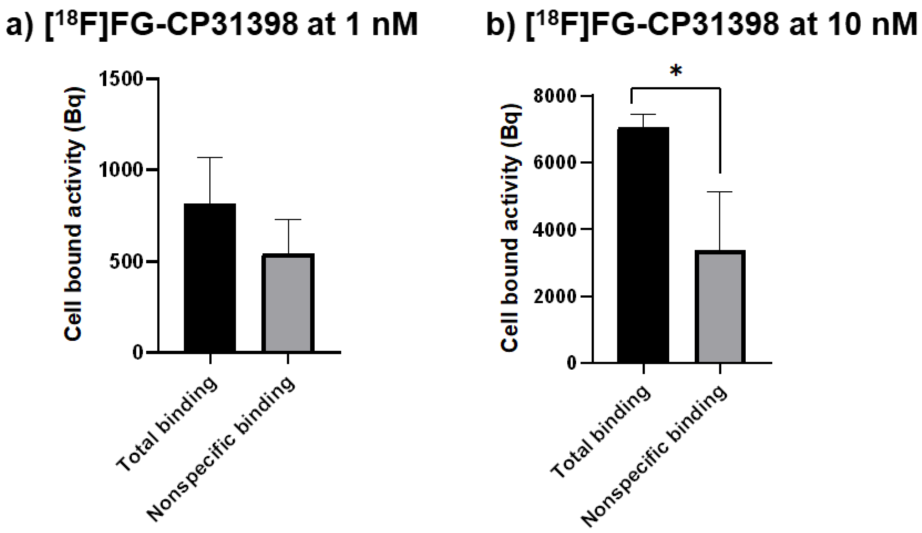

Figure S7. Binding experiments on HEK-293T cells transfected with the GFP-p53wt plasmid with an incubation of the cells at 1 nM (a) and 10 nM (b) of [^{18}F]**FG-CP31398** with (grey) or without (black) pre-saturation with a 100-fold excess of **CP31398**.

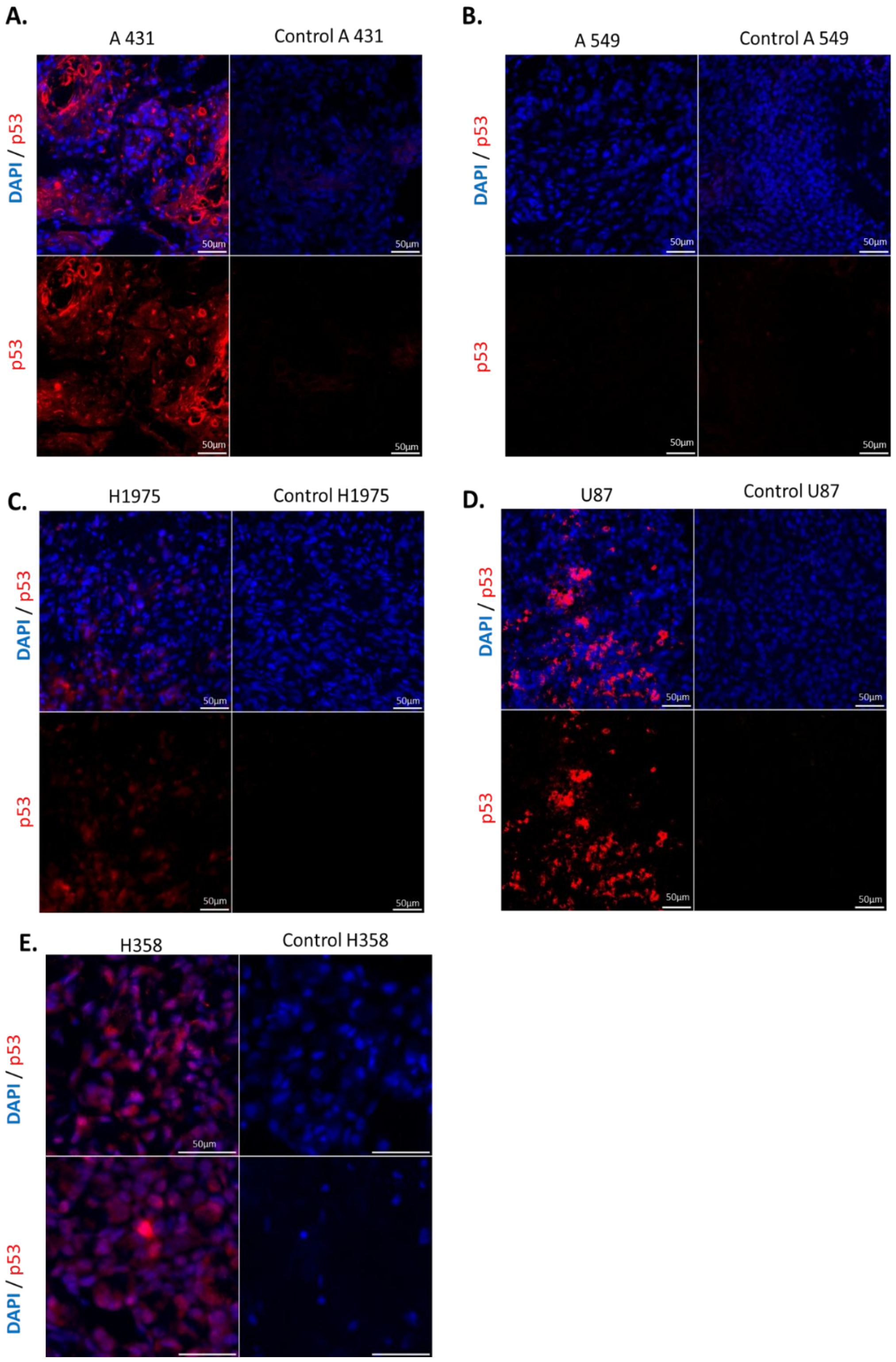

Figure S8. Immunofluorescence staining of tumor sections derived from xenografts of A431 (A), A549 (B), H1975 (C), U87 (D), and H358 (E) cell lines in mice. Sections were stained for p53 (red) and counterstained with DAPI (blue) to visualize cell nuclei.

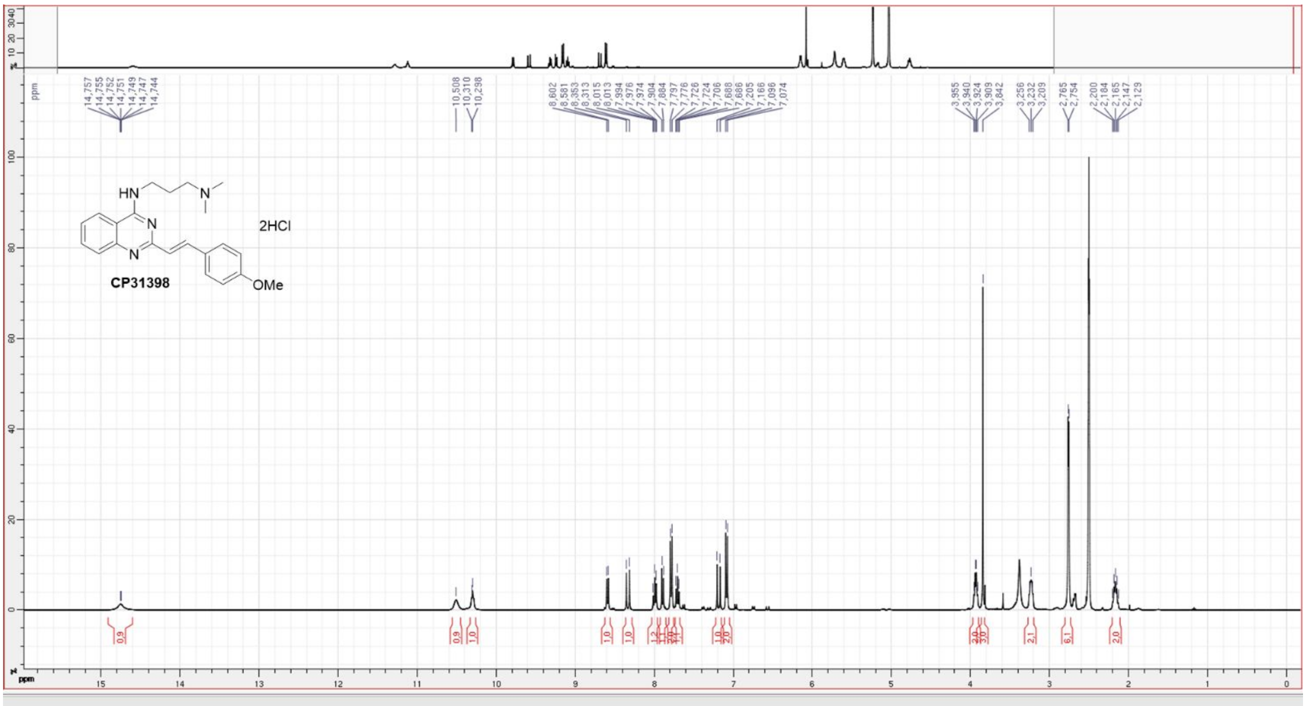

Figure S9. ^{1}H-NMR of compound **CP31398**.

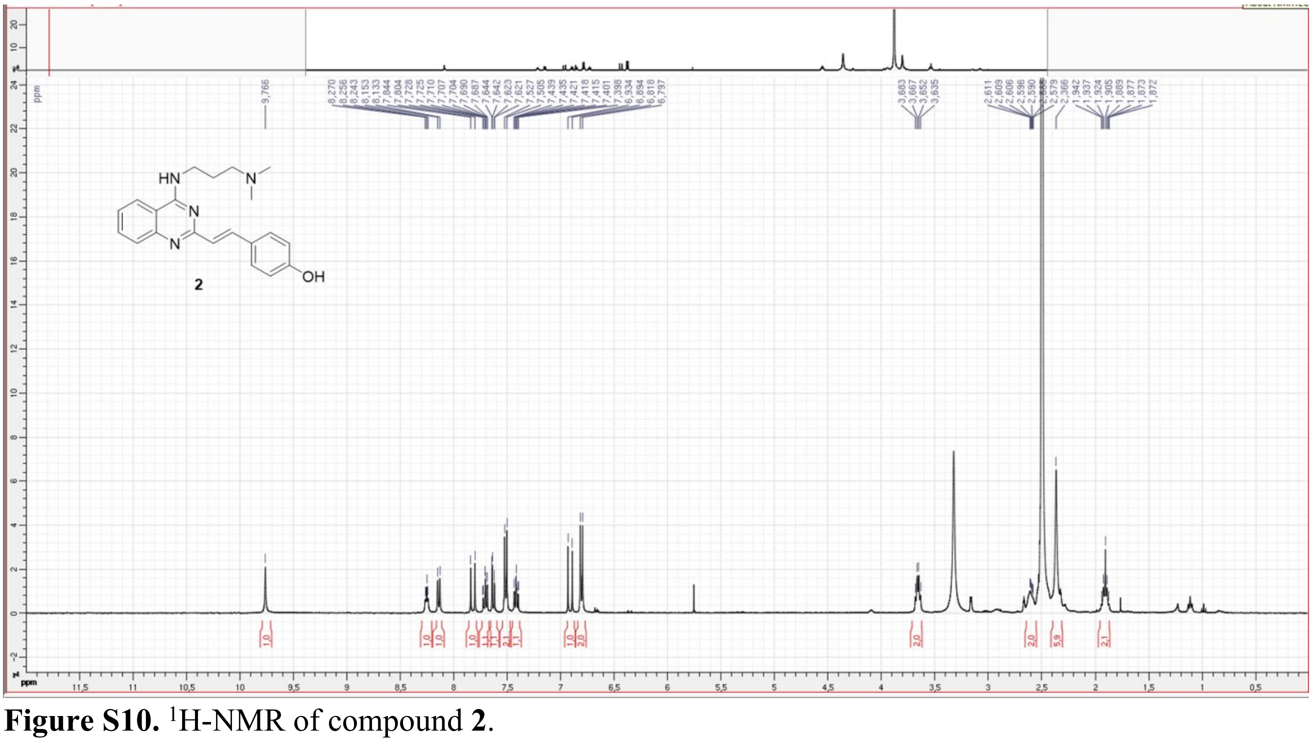

Figure S10. ^{1}H-NMR of compound **2**.

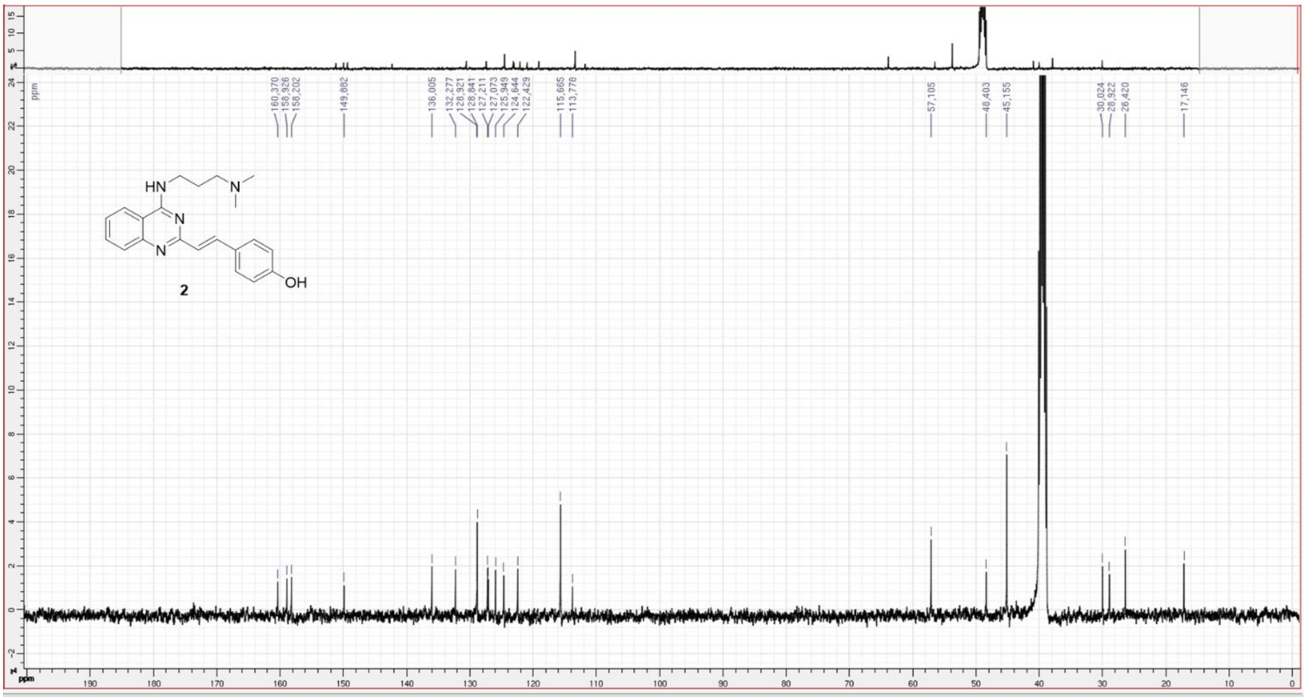

Figure S11. ^{13}C-NMR of compound **2**.

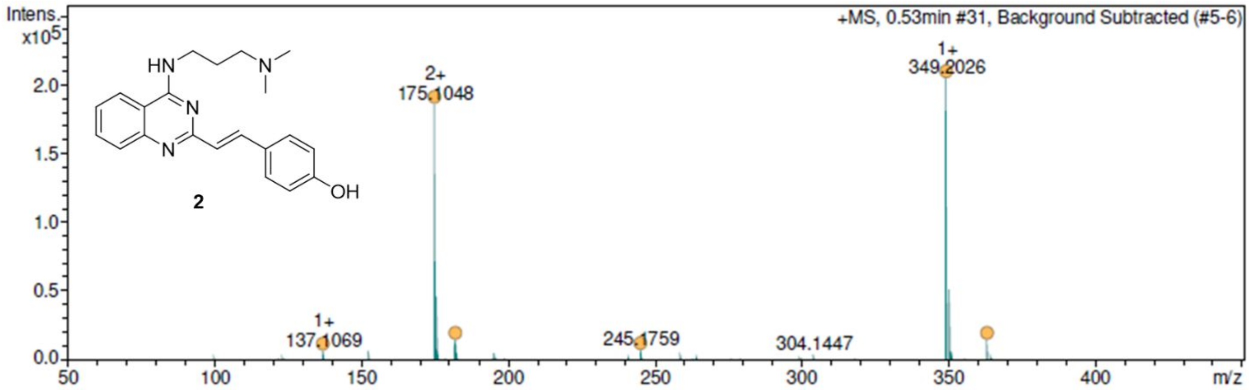

Figure S12. HRMS analysis of compound **2**.

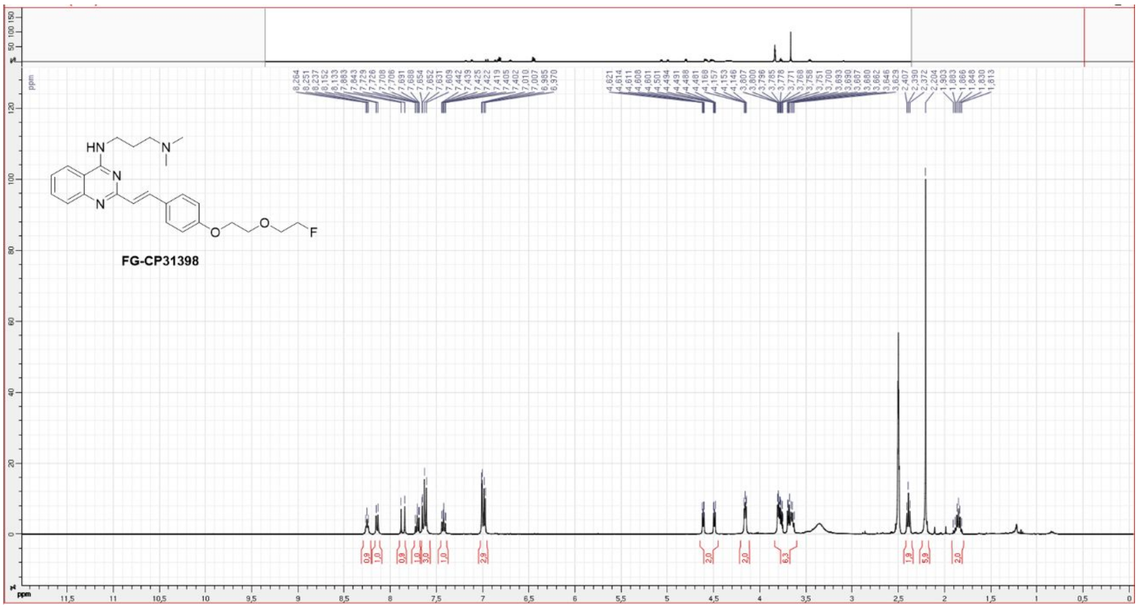

Figure S13. ^{1}H-NMR of compound **FG-CP31398**.

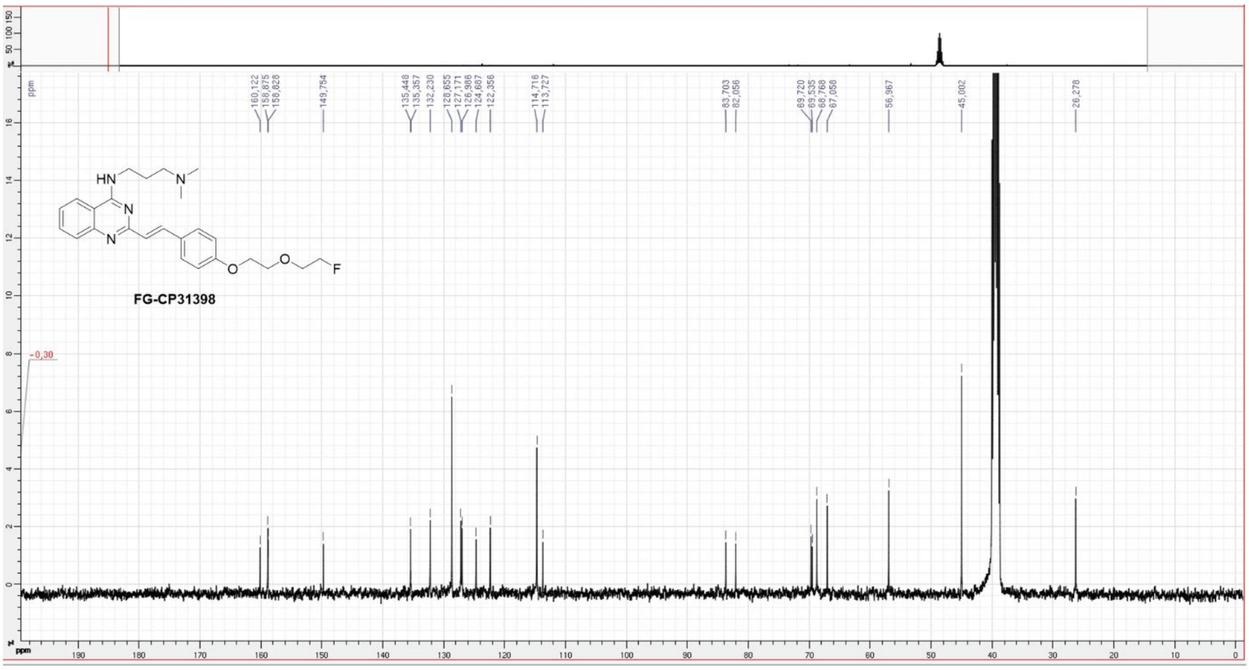

Figure S14. ^{13}C-NMR of compound **FG-CP31398**.

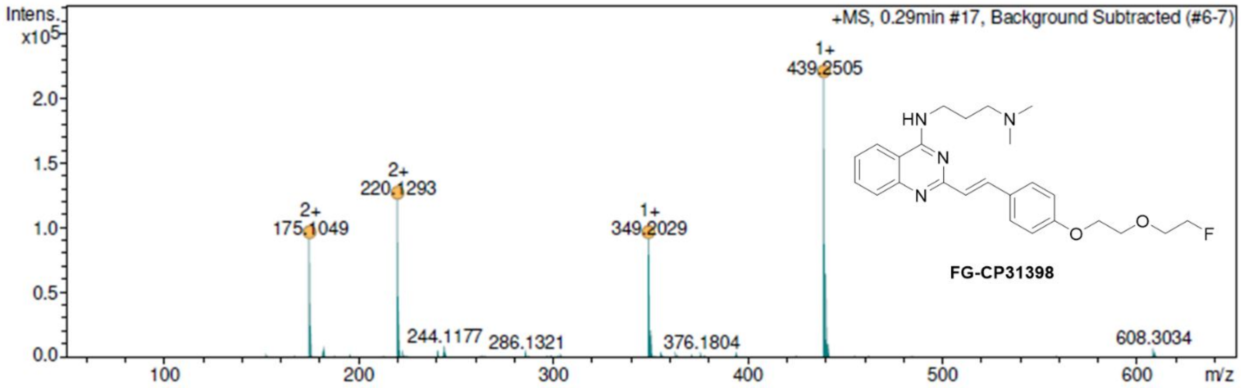

Figure S15. HRMS analysis of compound **FG-CP31398**.

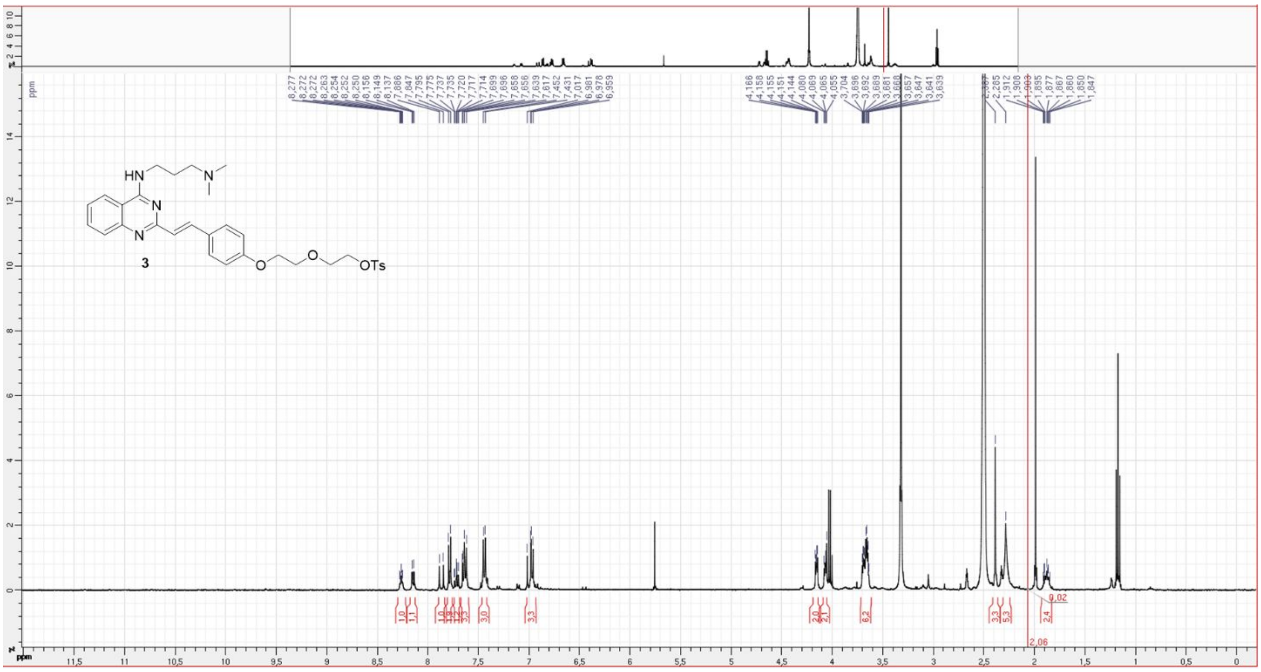

Figure S16. ^{1}H-NMR of compound **3**.

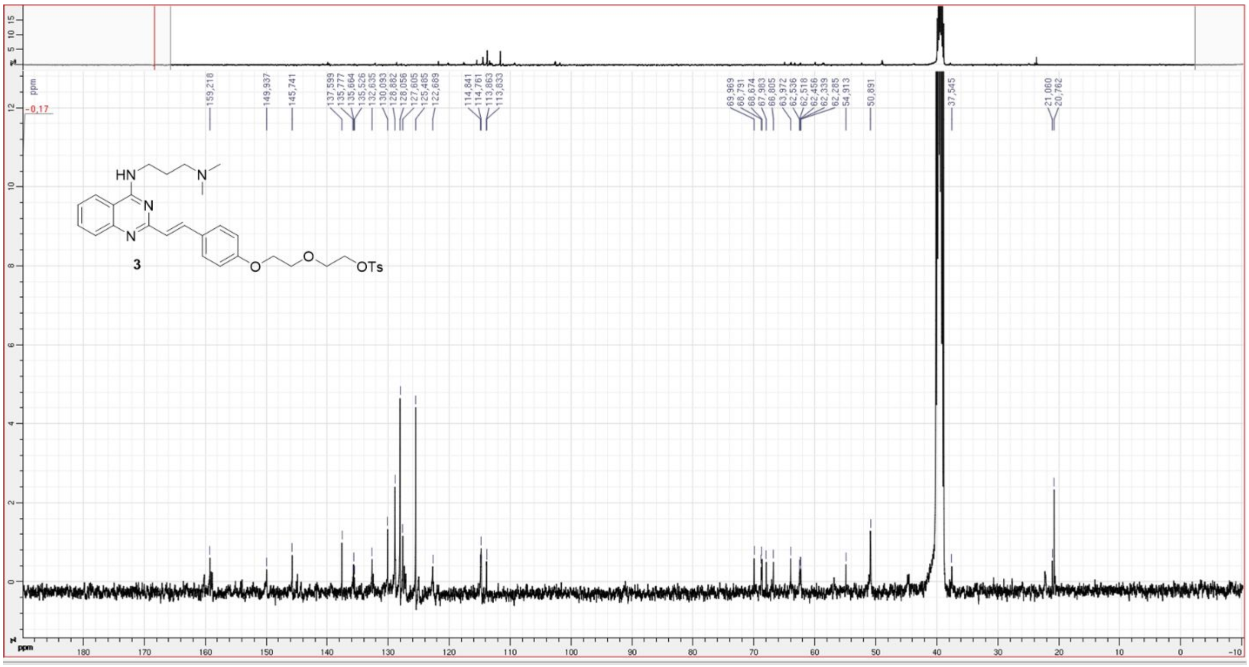

Figure S17. ^{13}C-NMR of compound **3**.

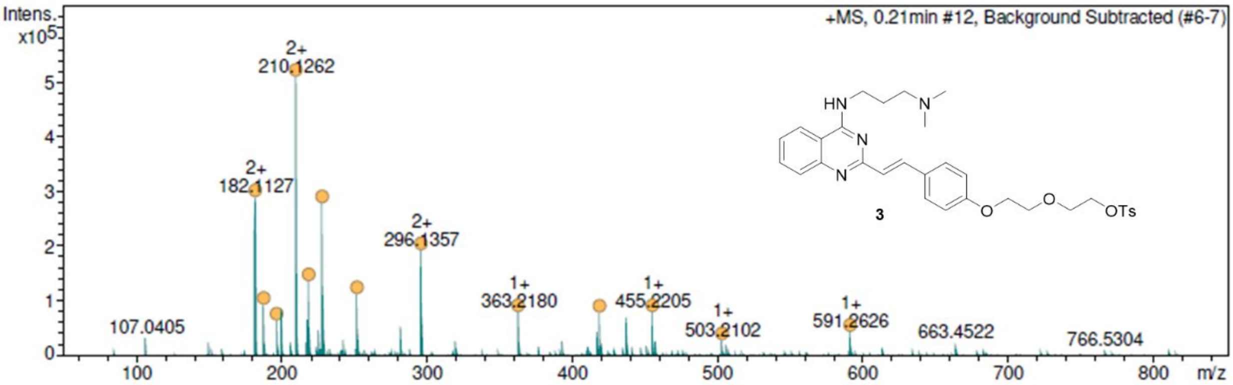

Figure S18. HRMS analysis of compound **3.**

Synthesis of compound F-CP31398 and its labeling precursor and attemps of radialabeling with fluorine-18

Compound **F-CP31398** bears a fluorine atom in position 7 of the quinazolinone scaffold which must be introduced at the beginning of the synthesis. Therefore, compound **S1** was synthesized in two steps from 2-amino-4-fluorobenzoic acid following a method of the literature (Scheme S1).[1] From compound **S1**, the same approach as for the synthesis of **CP31398** and inspired by Sutherland *et al.*[2] was realized to afford compound **F-CP31398** as a chlorhydrate salt in 5 steps and 33% overall yield.

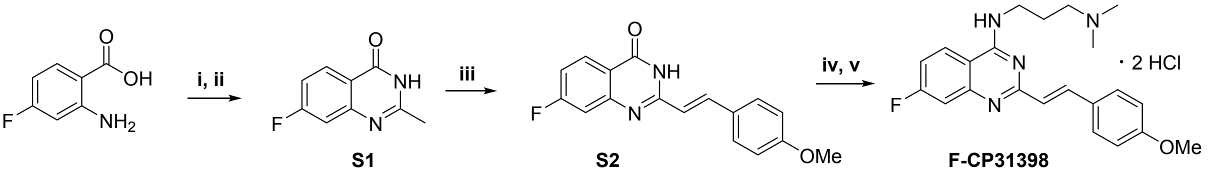

Scheme S1. Synthesis of compound **F-CP31398**. *Reagents and conditions:* i) Ac_2O, 120 °C, 3h; ii) NH_{3aq}, 120 °C, 4h, 66% over two steps; iii) *p*-anisaldehyde, AcOK, AcOH, 110 °C, 30h, 55%; iv) N^1,N^1-dimethylpropane-1,3-diamine, BOP, DBU, CH_3CN, r.t., 24h; v) HCl, MeOH, r.t., 5 min, 90% over two steps.

Considering the position of the fluorine atom on an electron-rich aromatic ring, we anticipated that radiofluorination with fluorine-18 could not be performed by standard aromatic nucleophilic substitution. Instead, copper-mediated Cham-Lam-like radiofluorination from trialkyltin precursors is a powerful approach to radiolabel electron-rich aromatic rings with fluorine-18.[3] From our own experience, trimethyltin precursors yield higher radiochemical conversion than other trialkyltin derivatives as far as copper-mediated fluorination is concerned, a result confirmed in the literature with tyrosine scaffolds.[4] As a result, we decided to synthesize a trimethyltin precursor for the radiofluorination of compound **F-CP31398**. Following the same approach as for **F-CP31398**, the bromine derivative **S4** was synthesized in two steps and 38% overall yield from commercially available 7-bromo-2-methylquinazolin-4(*3H*)-one (Scheme S2). Palladium-catalyzed stannylation reaction with hexamethylditin in presence of a Pd(0) catalyst afforded the desired precursor **S5** in 69% yield.

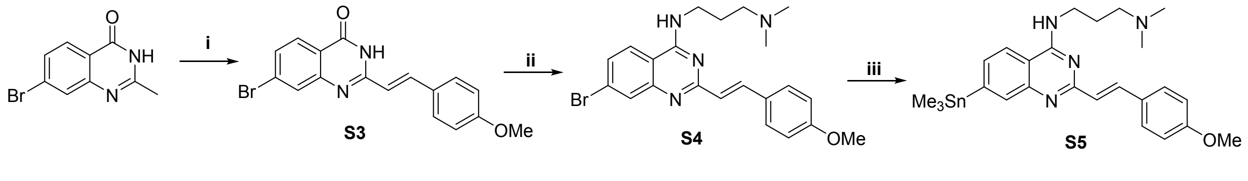

Scheme S2. Synthesis of the trimethylstananne precursor **S5**. *Reagents and conditions* : i) *p*-anisaldehyde, AcOK, AcOH, 110 °C, 30h, 75%; ii) *N¹,N¹*-dimethylpropane-1,3-diamine, BOP, DBU, CH_3CN, r.t., 24h; 51%; iii) Sn_2Me_6, $Pd(PPh_3)_4$, 1,4-dioxane, 100 °C, 18h, 69%.

The radiolabeling of **F-CP31398** was performed by copper-mediated Chan-Lam-like radiofluorination from the trimethyltin precursor **S5** (Scheme S3). Inspired by the conditions described by Makaravage *et al.,*[3] the radiofluorination was carried out with 4 mg (7.6 µmol) of **S5** and 2.5 equivalents (13 mg) of the commercially available $Cu(OTf)_2Py_4$ complex using a Trasis AllInOne synthesizer. Cyclotron-produced [^{18}F]fluoride was dried in the presence of potassium triflate to form K[^{18}F]F.

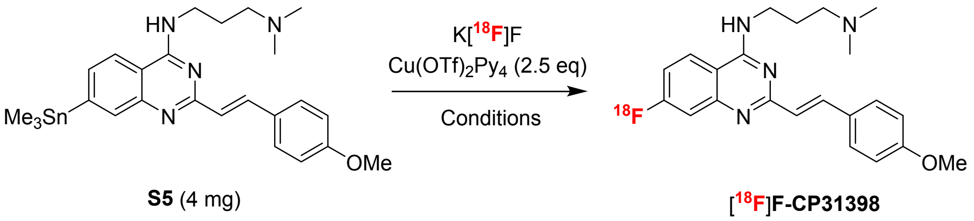

Scheme S3. Radiofluorination of precursor **S5** by copper-assisted radiofluorination.

Optimization of the reaction conditions are depicted in Table S1. The RCC was evaluated by radioHPLC of the crude reaction. Under the standard conditions of Makaravage *et al.* using dimethylacetamide (DMA) as solvent at 110 °C for 10 min,[3] no desired product could be observed by radioHPLC (Table S1, entry 1). A longer reaction time of 20 min resulted in the same observation (Table S1, entry 2). It has been described that 1,2-dimethyl-2-imidazolidinone (DMI) afforded higher yields than DMA when used as solvent for copper-assisted radiofluorination of arylstannane.[5] Unfortunately, performing the reaction in DMI at 110 °C for 20 min did not afford any [^{18}F]**F-CP31398** (Table S1, entry 3). With a higher temperature of 140 °C, traces of the desired radiotracer could be observed, together with the degradation of the major part of precursor **S5** (Table S1, entry 4). This observation prevented the use of higher temperatures. These results can be explained by the presence of an unprotected amine function which can drastically poisoned the copper-mediated radiofluorination.[6] From our experience on radiofluorination of trimethyltin precursors, changing other parameters like the stoichiometry of the copper complex, the elution conditions of the [^{18}F]fluoride or the quantity of precursor will not have any positive effect on the conversion. Facing the difficulty to radiolabel compound **F-CP31398**, we decided not to pursue further with this molecule for the *in vitro* evaluation of the radiotracers.

Table S1. Optimization of the radiofluorination of precursor **S5**. Reactions were performed with 2.5 eq of $Cu(OTf)_2Py_4$ complex on a AllInOne module using 4 mg of **S5**.

Entry[a]	Solvent	Time	Temperature	RCC[b]
**1**	DMA	10 min	110 °C	0 %
**2**	DMA	20 min	110 °C	0 %
**3**	DMI	20 min	110 °C	0 %
**4**	DMI	20 min	140 °C	<1 %

[a]Experiments were realized in duplicate

[b]The RCC was measured by radioHPLC as the ratio of the AUC of the [^{18}F]**F-CP31398** peak over the sum of the AUC of all peaks

7-fluoro-2-methylquinazolin-4(*3H*)-one (S1). Acetic anhydride (1.5 mL, 9.0 equiv.) was added on 2-amino-4-fluorobenzoic acid (250 mg, 1.0 equiv.) and the mixture was stirred at 120°C for 3h. After cooling to room temperature, the mixture was concentrated under vacuum and ammonia (28% v/v in water, 3 mL) was added. The mixture was stirred at 120°C for 4h. The precipitate formed was filtered and washed with cold water (15 mL) to afford **S1** as a light brown solid (190 mg, 66%). ^{1}H-NMR (DMSO-d_6, 400 MHz): δ 12.12 (s, 1H), 8.12 (dd, J = 8.9 Hz, J = 6.4 Hz, 1H), 7.31 (m, 2H), 2.35 (s, 3H) ppm. ^{13}C-NMR (DMSO-d_6, 100 MHz): δ 165.6 (d, J = 250 Hz), 161.0, 155.9, 151.1 (d, J = 13 Hz), 128.7 (d, J = 12 Hz), 117.6 (d, J = 2 Hz), 114.3 (d, J = 24 Hz), 111.6 (d, J = 22 Hz), 21.4 ppm. M.p. 280-285°C. LC/MS: t_R = 3.42 min.; 179.02 m/z $[M+H]^+$.

***(E)*-7-fluoro-2-(4-methoxystyryl)quinazolin-4(*3H*)-one (S2).** To a solution of **S1** (165 mg, 1.0 equiv.) in acetic acid (4 mL) were added dry potassium acetate (230 mg, 3.0 equiv.) and *p*-methoxybenzaldehyde (283 µL, 2.5 equiv.). The reaction was heated in a sealed tube at 110°C for 24h. The solution was cooled to 0 °C and the precipitate formed was filtered and washed with cold ethanol (5 mL) to afford **S2** (150 mg, 55 %) as a brown powder. ^{1}H-NMR (DMSO-d_6, 400 MHz): δ 12.35 (s, 1H), 8.15 (dd, J = 8.8 Hz, J = 6.4 Hz, 1H), 7.92 (d, J = 16.0 Hz, 1H), 7.62 (d, J = 8.7 Hz, 2H), 7.40 (dd, J = 10.2 Hz, J = 2.5 Hz, 1H), 7.32 (td, J = 8.8 Hz, J = 2.5 Hz, 1H), 7.04 (d, J = 8.8 Hz, 2H), 6.85 (d, J = 16.0 Hz, 1H), 3.82 (s, 3H) ppm. ^{13}C-NMR (DMSO-d_6, 100 MHz): δ 164.4, 161.5, 160.7, 153.6, 151.3, 138.6, 129.3 (2C), 128.9, 127.5, 118.5, 118.0 (2C), 114.5 (2C), 111.8, 55.3 ppm. M.p. 285-288°C. LC/MS: t_R = 4.43 min.; 297.04 m/z $[M+H]^+$.

***(E)-N¹*-(7-fluoro-2-(4-methoxystyryl)quinazolin-4-yl)-*N³,N³*-dimethylpropane-1,3-diamine dihydrochloride (F-CP31398).** To a solution of **S2** (130 mg, 1.0 equiv.) in CH_3CN (2.5 mL) were added BOP (234 mg, 1.2 equiv.) and DBU (80 µL, 1.2 equiv.). The reaction mixture was stirred for 10 min under argon at room temperature and 3-(dimethylamino)-1-propylamine (73 µL, 1.5 equiv.) was added. The solution was stirred for 24h at room temperature under argon. The mixture was concentrated to dryness and HCl (1.25 M in MeOH, 2 mL) was added followed by EtOAc (5 mL) and the precipitate formed was filtered and washed with cold CH_3CN (5 mL) to afford **F-CP31398** (185 mg, 90%) as a yellow solid. ^{1}H-NMR (DMSO-d_6, 400 MHz): δ 8.55 (dd, *J* = 9.0 Hz, *J* =5.8 Hz, 1H), 8.05 (m, 2H), 7.71 (m, 2H), 7.45 (m, 3H), 7.03 (m, 2H), 3.83 (m, 5H), 3.22 (t, *J* = 7.4 Hz, 2H), 2.76 (s, 6H), 2.14 (m, 2H) ppm. ^{13}C-NMR (DMSO-d_6, 100 MHz): δ 166.2, 163.7, 161.0, 159.0 (2C), 144.0 (d, *J* = 230 Hz), 129.9 (2C), 127.5, 127.3, 124.4, 119.1, 114.5 (2C), 110.1, 109.6, 55.3, 54.4, 53.4, 42.0 (2C), 23.6 ppm. M.p. 197-200°C. HR-ESI(+)-MS m/z calcd for $C_{22}H_{26}FN_4O$: 381.2091 ; found 381.2087 $[M+H]^+$ (see Figures S19 to S21 for NMR and HRMS analysis).

***(E)*-7-bromo-2-(4-methoxystyryl)quinazolin-4(*3H*)-one (S3).** To a solution of 7-bromo-2-methylquinazolin-4(*3H*)-one (400 mg, 1.0 equiv.) in acetic acid (8 mL) were added dry potassium acetate (410 mg, 3.0 equiv.) and *p*-methoxybenzaldehyde (0.5 mL, 2.5 equiv.). The reaction was heated at 110°C in a sealed tube for 24h. The solution was cooled to 0 °C and the precipitate formed was filtered and rinsed with cold ethanol (10 mL) to afford S3 (445 mg, 75 %) as a white powder. ^{1}H-NMR (DMSO-d_6, 400 MHz): δ 7.98 (dd, *J* = 8.5 Hz, *J* = 2.2 Hz, 1H), 7.90 (d, *J* = 16.8 Hz, 1H), 7.80 (m, 1H), 7.59 (m, 3H), 7.02 (d, *J* = 8.8 Hz, 2H), 6.84 (d, *J* = 16.8 Hz, 1H), 3.81 (s, 3H) ppm. M.p. 215-220°C. LC/MS: t_R = 4.82 min.; 359.77 m/z $[M+H]^+$. ^{13}C-NMR could not be performed because of the insolubility of the product.

***(E)-N¹*-(7-bromo-2-(4-methoxystyryl)quinazolin-4-yl)-*N³,N³*-dimethylpropane-1,3-diamine (S4).** To a solution of **S3** (445 mg, 1.0 equiv.), in CH_3CN (8 mL) were added BOP (720 mg, 1.3 equiv.) and DBU (248 µL, 1.3 equiv.). The reaction mixture was stirred for 10 min under argon at room temperature. Then, 3-(dimethylamino)-1-propylamine (243 µL, 1.5 equiv.) was added and the solution was stirred for 24h at room temperature under argon. The precipitate formed was filtered and washed with CH_3CN (10 mL) to afford **S4** (280 mg, 51%) as a white solid. ^{1}H-NMR (DMSO-d_6, 400 MHz): δ 8.40 (b, 1H), 8.12 (d, *J* = 8.8 Hz, 1H), 7.87 (m, 2H), 7.64 (m, 3H), 6.99 (m, 3H), 3.80 (s, 3H), 3.69 (dd, *J* = 12.3 Hz, *J* =6.4, Hz, 2H), 3.10

(t, J = 7.5 Hz, 2H), 2.73 (s, 6H), 2.03 (m, 2H) ppm. ^{13}C-NMR (DMSO-d_6, 100 MHz): δ 161.4, 159.9, 158.9, 151.2, 136.5, 129.2, 129.0 (2C), 128.5, 127.7, 126.6, 125.9, 124.8, 114.3 (2C), 112.8, 57.0, 55.2, 45.2 (2C), 40.1, 26.4 ppm. M.p. 145-151°C. LC/MS: t_R = 5.48 min.; 443.13 m/z $[M+H]^+$.

(E)-N^1^-(2-(4-methoxystyryl)-7-(trimethylstannyl)quinazolin-4-yl)-N^1^,N^1^-dimethylpropane-1,3-diamine (S5). To a flame dried tube was added S4 (100 mg, 1.0 equiv.) and 1,4-dioxane (1 mL). The mixture was degassed for 10 min and tetrakistriphenylphosphine palladium (12 mg, 0.1 equiv.) and hexamethylditin (96 µL, 2.0 equiv.) were added. The mixture was stirred at 110 °C for 15h. Upon cooling to room temperature, the reaction mixture was concentrated under vacuum and the residue was purified on flash chromatography using DCM/MeOH/NH_4OH 9/1/0.1 v/v/v as eluent to afford **S5** (86 mg, 69%) as a yellow gum. ^{1}H-NMR (DMSO-d_6, 400 MHz): δ 8.23 (t, J = 5.4 Hz, 1H), 8.06 (d, J = 8.0 Hz, 1H), 7.85 (d, J = 15.9 Hz, 1H), 7.76 (s, 1H), 7.62 (d, J = 8.7 Hz, 2H), 7.53 (dd, J = 8.1 Hz, J =1.0 Hz, 1H), 7.0 (m, 3H), 3.80 (s, 3H), 3.66 (q, J = 6.6 Hz, 2H), 2.36 (t, J = 7.1 Hz, 2H), 2.19 (s, 6H), 1.84 (q, J = 7.1 Hz, 2H), 0.34 (t, J = 28 Hz, 9H) ppm. ^{13}C-NMR (DMSO-d_6, 100 MHz): δ 160.1, 159.8, 159.1, 148.9, 148.3, 135.6, 135.0, 131.6, 128.8 (2C), 128.7, 127.0, 121.3, 114.4 (2C), 113.6, 56.3, 55.2, 44.0 (2C), 38.4, 25.5, - 9.25 (3C) ppm. HR-ESI(+)-MS m/z calcd for $C_{25}H_{35}N_4OSn$: 527.1828; found 527.1828 $[M+H]^+$ (see Figures S22 to S24 for NMR and HRMS analysis).

*Radiolabeling of [^{18}F]**F-CP31398** with fluorine-18*

All reactions were carried out using a AllInOne module (Trasis, Belgium). No carrier-added [^{18}F]fluoride ion (10-20 GBq) was produced *via* the ^{18}O(p,n)^{18}F nuclear reaction by irradiation of a 2 mL [^{18}O]water (> 97% enriched, Rotem, Israël) target with an IBA Cyclone-18/9 (IBA, Belgium) cyclotron. [^{18}F]F^- was trapped on an ion exchange resin QMA light (Waters, USA) and eluted in the reactor using a mixture of an aqueous solution of potassium triflate (10 mg/mL, 450 µL, 23.9 µmol), an aqueous solution of potassium carbonate (1 mg/mL, 50 µL, 0.4 µmol) and CH_3CN (500 µL). The resulting complex was dried upon heating 120 °C for 7 min under vacuum. A solution of **S5** (4 mg, 7.6 µmol) and $Cu(OTf)_2Py_4$ (12 mg, 17.8 µmol) in DMA or DMI (500 µL) was added and the mixture was heated at 110 °C or 140 °C for 10 or 20 min. Upon cooling to room temperature, the crude was diluted in H_2O/CH_3CN (7/3 v/v, 2mL). The crude product was analyzed by HPLC according to the general procedure of quality control using a mixture of H_2O/CH_3CN (7/3 v/v) as eluent and UV detection at 254 nm.

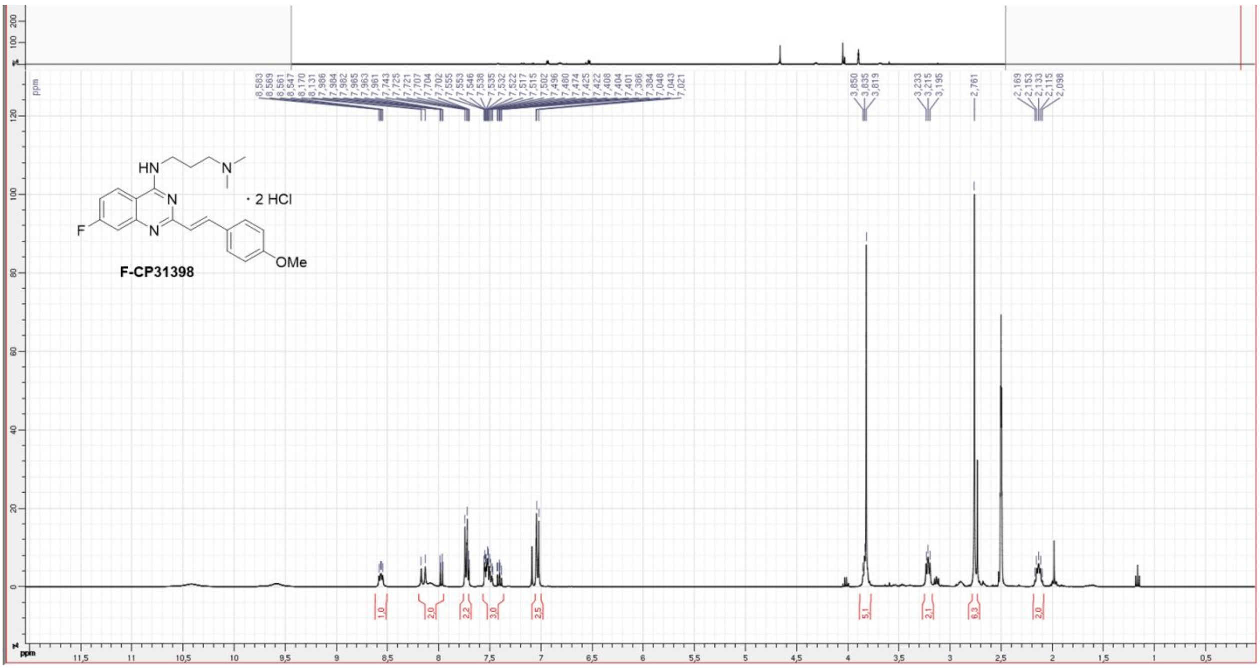

Figure S19. ^{1}H-NMR of compound **F-CP31398**.

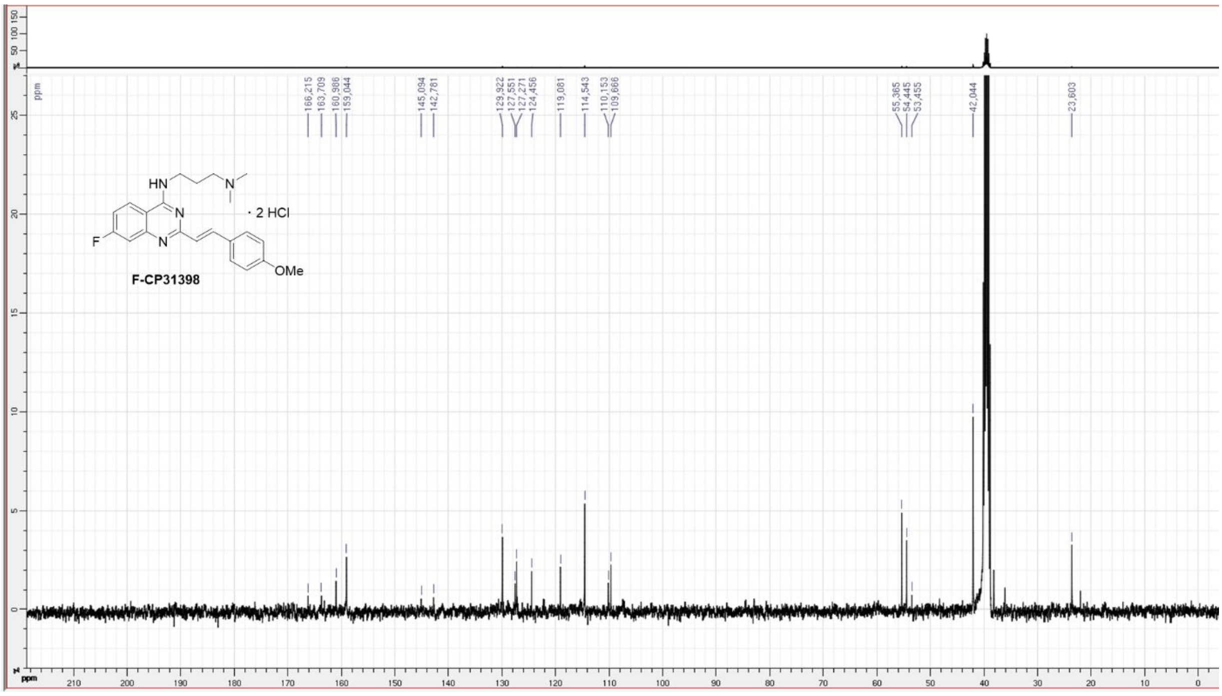

Figure S20. ^{13}C-NMR of compound **F-CP31398**.

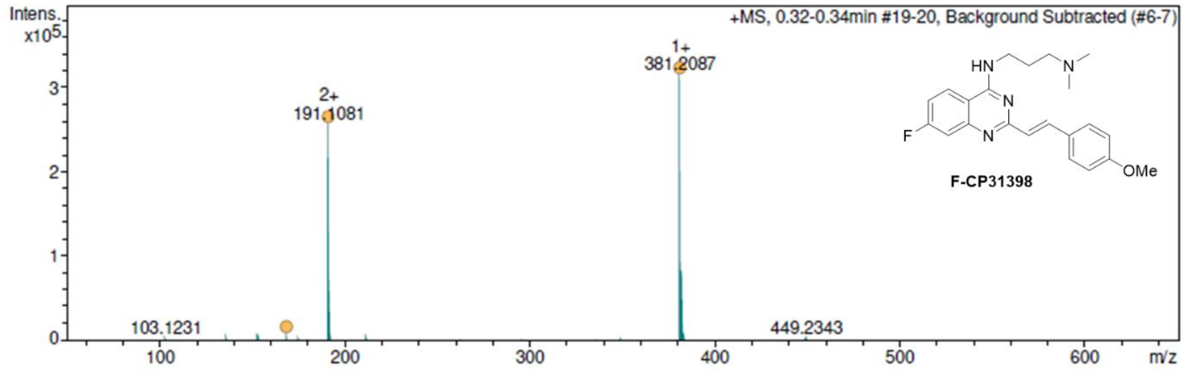

Figure S21. HRMS analysis of compound **F-CP31398.**

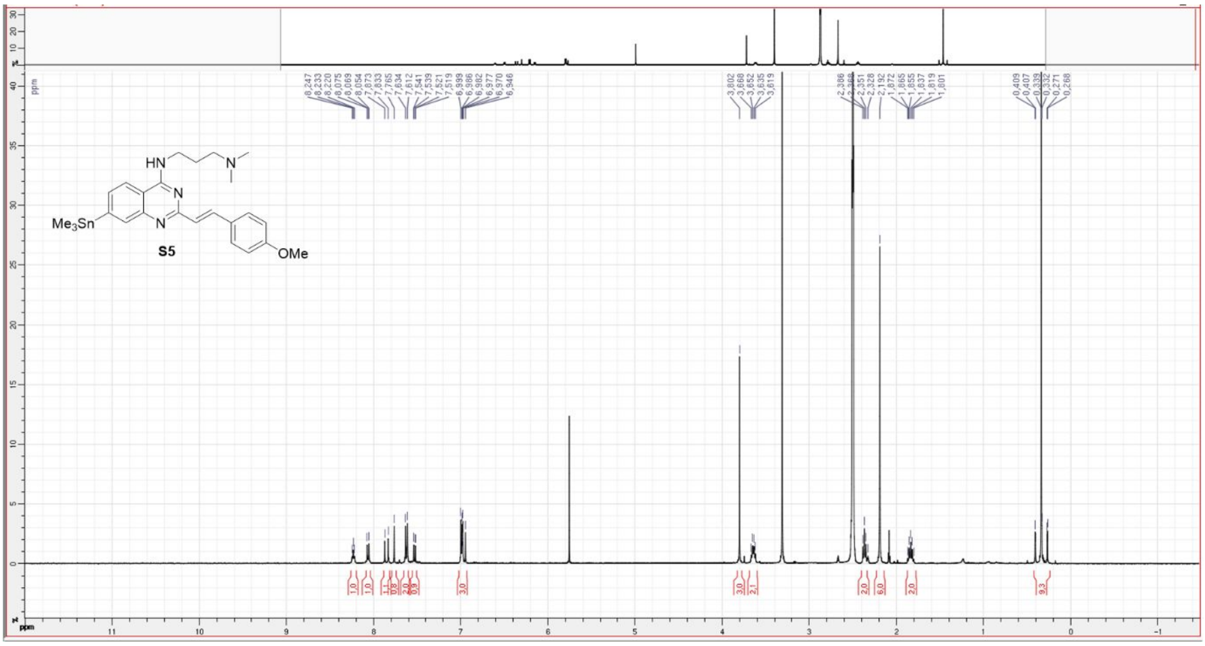

Figure S22. ^{1}H-NMR of compound **S5**.

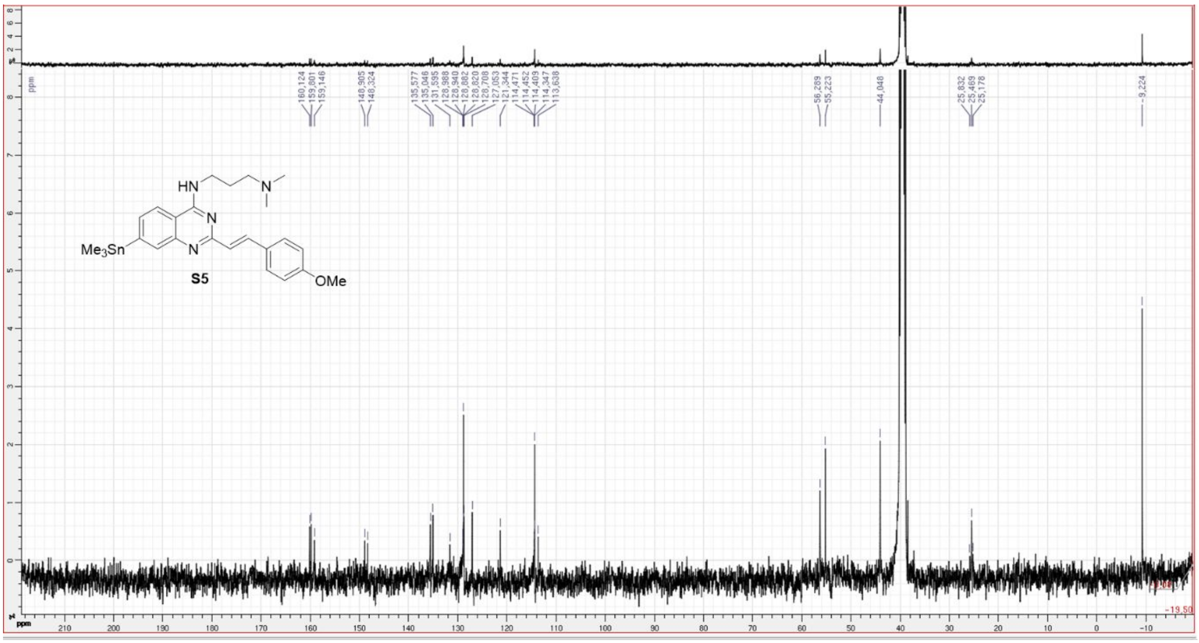

Figure S23. ^{13}C-NMR of compound **S5**.

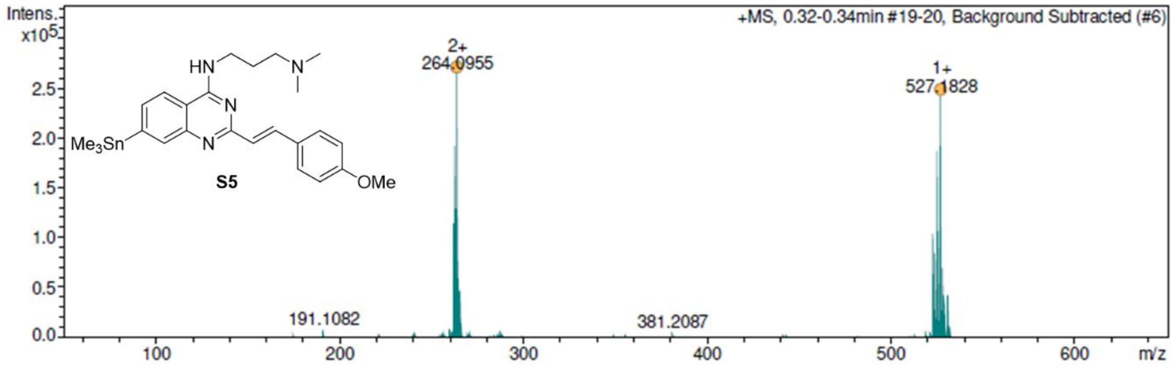

Figure S24. HRMS analysis of compound **S5.**

References

(1) Che, T.; Chen, S.-B.; Tu, J.-L.; Wang, B.; Wang, Y.-Q.; Zhang, Y.; Wang, J.; Wang, Z.-Q.; Zhang, Z.-P.; Ou, T.-M.; Zhao, Y.; Tan, J.-H.; Huang, Z.-S. Discovery of Novel Schizocommunin Derivatives as Telomeric G-Quadruplex Ligands That Trigger Telomere Dysfunction and the Deoxyribonucleic Acid (DNA) Damage Response. *J. Med. Chem.* **2018**, *61* (8), 3436–3453. https://doi.org/10.1021/acs.jmedchem.7b01615.

(2) Sutherland, H. S.; Hwang, I. Y.; Marshall, E. S.; Lindsay, B. S.; Denny, W. A.; Gilchrist, C.; Joseph, W. R.; Greenhalgh, D.; Richardson, E.; Kestell, P.; Ding, A.; Baguley, B. C. Therapeutic Reactivation of Mutant p53 Protein by Quinazoline Derivatives. *Invest. New Drugs* **2012**, *30* (5), 2035–2045. https://doi.org/10.1007/s10637-011-9744-z.

(3) Makaravage, K. J.; Brooks, A. F.; Mossine, A. V.; Sanford, M. S.; Scott, P. J. H. Copper-Mediated Radiofluorination of Arylstannanes with [18F]KF. *Org. Lett.* **2016**, *18* (20), 5440–5443. https://doi.org/10.1021/acs.orglett.6b02911.

(4) Chao, M. N.; Chezal, J.-M.; Debiton, E.; Canitrot, D.; Witkowski, T.; Levesque, S.; Degoul, F.; Tarrit, S.; Wenzel, B.; Miot-Noirault, E.; Serre, A.; Maisonial-Besset, A. A Convenient Route to New (Radio)Fluorinated and (Radio)Iodinated Cyclic Tyrosine Analogs. *Pharmaceuticals* **2022**, *15* (2), 162. https://doi.org/10.3390/ph15020162.

(5) Hoffmann, C.; Kolks, N.; Smets, D.; Haseloer, A.; Gröner, B.; Urusova, E. A.; Endepols, H.; Neumaier, F.; Ruschewitz, U.; Klein, A.; Neumaier, B.; Zlatopolskiy, B. D. Next Generation Copper Mediators for the Efficient Production of 18F-Labeled Aromatics. *Chem. – Eur. J.* **2023**, *29* (2), e202202965. https://doi.org/10.1002/chem.202202965.

(6) Taylor, N. J.; Emer, E.; Preshlock, S.; Schedler, M.; Tredwell, M.; Verhoog, S.; Mercier, J.; Genicot, C.; Gouverneur, V. Derisking the Cu-Mediated 18F-Fluorination of Heterocyclic Positron Emission Tomography Radioligands. *J. Am. Chem. Soc.* **2017**, *139* (24), 8267–8276. https://doi.org/10.1021/jacs.7b03131.
